# Decoding lipid metabolic reprogramming in pancreatic ductal adenocarcinoma: from tumor–microenvironment crosstalk to therapeutic resistance

**DOI:** 10.3389/fonc.2026.1977031

**Published:** 2026-10-26

**Authors:** Xingyu Liu, Zitong Ye, Liuyang Wang, Yubo Chi, Tongjia Chu, Ziyu Liu, Shanxiang Yu, Haocheng Yang, Xinyue Yin, Jian Zhang, Alfred Wei Chieh Kow, Huan Liu

**Affiliations:** 1 Department of Hepatobiliary and Pancreatic Surgery, General Surgery Center, The First Hospital of Jilin University, Changchun, Jilin, China; 2 The First Hospital of Jilin University, Changchun, Jilin, China; 3 Department of Colorectal & Anal Surgery, General Surgery Center, The First Hospital of Jilin University, Changchun, Jilin, China; 4 Department of Pancreatic Surgery, Fudan University Shanghai Cancer Center, Shanghai, China; 5 Jilin University School of Public Health, Changchun, Jilin, China; 6 Division of Hepatobiliary & Pancreatic Surgery, Department of Surgery, National University Hospital Singapore, Singapore, Singapore; 7 Department of Surgery, Yong Loo Lin School of Medicine, National University of Singapore, Singapore, Singapore; 8 Division of Surgical Oncology, National University Cancer Institute Singapore (NCIS), National University Health System, Singapore, Singapore; 9 National University Digestive Center (NUCD), National University Health System Singapore, Singapore, Singapore

**Keywords:** cholesterol metabolism, fatty acid metabolism, lipid metabolism, metabolic plasticity, pancreatic ductal adenocarcinoma, therapeutic resistance, tumor microenvironment

## Abstract

Pancreatic ductal adenocarcinoma (PDAC) is characterized by a nutrient-poor, hypoxic, and highly desmoplastic microenvironment that imposes substantial metabolic stress on malignant cells. Under these unfavorable conditions, lipid metabolism reprogramming provides cancer cells with the flexibility needed to maintain growth and survive therapeutic stress. Recent advances have uncovered new links between lipid metabolic reprogramming, the tumor microenvironment, and therapeutic resistance in PDAC, highlighting the need for an updated synthesis of this rapidly evolving field. In this review, we focus on the major tumor-intrinsic alterations in fatty acid and cholesterol–mevalonate metabolism, including changes in lipid synthesis, uptake, desaturation, storage, and oxidative utilization. These processes support membrane homeostasis, oncogenic signaling, and redox control, thereby linking lipid handling directly to tumor progression and treatment adaptation. We further review how these pathways are reshaped by the tumor microenvironment, where cancer-associated fibroblasts, immune cells, adipocytes, and neural-associated cells influence lipid availability, trafficking, and signaling. These reciprocal interactions can shape stromal plasticity, immune suppression, invasion, metastasis, and treatment response. We also evaluate current strategies targeting lipid metabolism and discuss why their clinical translation remains limited by metabolic compensation, tumor heterogeneity, drug specificity, and systemic toxicity. Overall, the available evidence suggests that lipid metabolism in PDAC is better understood as a dynamic, interconnected adaptive network rather than a set of isolated metabolic pathways. This perspective supports biomarker-guided combination strategies that target context-dependent lipid dependencies and treatment-induced metabolic adaptation, rather than relying on inhibition of a single pathway.

## 1 Introduction

The global burden of pancreatic ductal adenocarcinoma (PDAC) has increased steadily over the past three decades and is projected to continue rising over the coming decades (1, 2). According to the latest GLOBOCAN 2024 statistics, approximately 531, 000 new cases and 491, 000 deaths occur globally each year, ranking pancreatic cancer eleventh in cancer incidence but sixth in cancer mortality and accounting for approximately 5% of all cancer-related deaths (3). One major reason for this poor outcome is the difficulty of early detection. Early-stage PDAC is often asymptomatic or presents with subtle and nonspecific symptoms, such as abdominal or back pain, dyspepsia, nausea, and weight loss, while jaundice may occur particularly in tumors involving the pancreatic head (4, 5). As a result, most patients already have locally advanced, unresectable, or metastatic disease at the time of diagnosis, and only a small proportion are eligible for upfront surgical resection (6). For patients with advanced disease, the median survival with treatment is generally around 10–12 months, whereas untreated patients survive only approximately 5–6 months (4). In addition, PDAC also exhibits aggressive biological features, including rapid disease progression, early metastasis, marked heterogeneity, and resistance to various therapeutic strategies (7). Together, these clinical challenges underscore the importance of understanding the biological mechanisms underlying PDAC progression and treatment resistance.

Beyond its aggressive clinical behavior, PDAC also has a distinctive tumor microenvironment (TME) characterized by extensive desmoplasia and poor vascular perfusion, which limit the delivery of oxygen and nutrients to tumor cells (8). To survive under these conditions, PDAC cells can adjust their metabolic programs in response to nutrient availability and cellular stress (9). The contribution of lipids to tumor metabolism has received growing attention in recent years. Lipids can provide an alternative source of energy when other nutrients are insufficient (10, 11). They also engage in membrane synthesis and activate signaling pathways that regulate cancer cell growth and migration (12). Cancer cells often show an increased demand for lipids and cholesterol, which can be met through enhanced uptake of extracellular lipids and lipoproteins as well as increased *de novo* lipid synthesis (13). These changes support tumor development and progression and may also contribute to abnormal lipid accumulation within the TME (13). Therefore, a deeper insight into lipid metabolic reprogramming may improve our understanding of PDAC and help identify potential biomarkers and therapeutic targets.

In this review, we summarize the major alterations in lipid metabolism that contribute to PDAC progression and discuss current and emerging strategies targeting these metabolic pathways. We also examine the metabolic interactions between tumor cells and the surrounding TME, providing an integrated view of lipid metabolic reprogramming and its potential therapeutic relevance in PDAC.

## 2 Intrinsic lipid metabolic reprogramming in PDAC

Lipid metabolic reprogramming plays an important role in PDAC biology (14, 15). Rather than relying on a single lipid source or pathway, PDAC cells coordinately regulate endogenous lipid synthesis, extracellular lipid uptake, fatty acid (FA) desaturation, lipid droplet (LD) storage, and oxidative utilization. Together, these processes support membrane formation, oncogenic signaling, redox homeostasis, and help cancer cells survive under nutrient limitation, hypoxia, and therapeutic stress (16–19). Importantly, these pathways do not operate independently. FA and cholesterol synthesis both depend on citrate-derived acetyl-CoA, whereas LDs store excess FAs and cholesteryl esters (CEs), reducing lipotoxicity and endoplasmic reticulum (ER) stress while preserving a lipid pool that can be mobilized when needed (16, 17, 20). When endogenous lipid synthesis or oxygen-dependent desaturation is limited, PDAC cells can also increase their use of extracellular or stromal-derived lipids (21).

Among the various lipids involved in cancer metabolism, this section mainly focuses on FA and cholesterol metabolism. FAs provide essential substrates for membrane phospholipid (PL) and triglyceride (TAG) synthesis, protein lipidation, redox regulation, and energy production (11, 21, 22). The mevalonate pathway supports membrane structure through cholesterol synthesis and generates non-sterol isoprenoid intermediates that regulate oncogenic signaling proteins (23, 24). Taken together, these two interrelated metabolic pathways encompass several key lipid-dependent processes that support PDAC progression and enable it to adapt to nutritional restriction and therapeutic stress (24). Other lipid classes are discussed when they act as downstream products, storage forms, or functional mediators of these pathways. Therefore, the following sections will explore the main enzymatic nodes in FA and cholesterol metabolism and their roles in PDAC growth, stress tolerance, treatment resistance, and possible metabolic targets.

### 2.1 Fatty acid metabolic reprogramming

FA metabolism in PDAC is characterized not by a uniform increase or decrease in total FA abundance, but by coordinated remodeling of FA sources, composition, and metabolic fate. PDAC cells sustain FA availability through both *de novo* synthesis and extracellular lipid acquisition (25, 26).

*De novo* FA synthesis is frequently engaged in PDAC. Increased fatty acid synthase (FASN) expression and the dependence of PDAC growth on Acetyl-CoA carboxylase (ACC) activity support the importance of this pathway (22, 27). Consistent with this dependence, FASN overexpression has been linked to chemoresistance, whereas genetic or pharmacological inhibition of FASN has enhanced gemcitabine sensitivity and suppressed tumor growth in preclinical models (28, 29).

In parallel, extracellular lipid acquisition provides a complementary source of FAs. PDAC cells acquire extracellular FAs through transporter-associated pathways involving CD36 and members of the solute carrier family 27/fatty acid transport protein (SLC27/FATP) family, including SLC27A1/FATP1 (30, 31). CD36 is upregulated in PDAC and has been associated with poor clinical outcomes and gemcitabine resistance. Its inhibition restored chemosensitivity *in vitro* and reduced lipid-supported tumor survival in neoadjuvant-treated orthotopic models (30, 32). Macropinocytosis may also contribute to extracellular FA acquisition, linking nutrient scavenging to lipid availability (33). This uptake program may become particularly important when endogenous lipid synthesis is insufficient or when tumors experience nutrient limitation.

Beyond determining the source of FAs, PDAC cells must also maintain an appropriate balance between saturated and unsaturated FAs, linking FA acquisition to desaturation-dependent lipid homeostasis. Stearoyl-CoA desaturase 1 (SCD1) mediated FA desaturation supports PDAC cell survival and ferroptosis resistance across multiple regulatory conditions (34, 35). However, because SCD1-dependent desaturation requires oxygen, the hypoxic and poorly perfused PDAC microenvironment can constrain endogenous unsaturated FA production, thereby increasing reliance on extracellular or stromal-derived unsaturated FAs (21, 36). Therefore, PDAC cells preserve FA homeostasis through coordinated regulation of synthesis, uptake, and desaturation pathways, generating context-dependent metabolic vulnerabilities that may be therapeutically exploited.

#### 2.1.1 *De novo* fatty acid synthesis

##### 2.1.1.1 Fatty acid synthase: FASN

FASN is the central multienzyme complex responsible for *de novo* FA synthesis. Using acetyl-CoA as a primer and seven malonyl-CoA molecules as two-carbon donors, FASN generates the 16-carbon saturated FA palmitate, which serves as a precursor for more complex FAs, membrane lipids, and protein palmitoylation (37, 38).

Immunohistochemical analyses detected FASN overexpression in most primary PDAC and intraductal papillary mucinous neoplasms (IPMNs). In IPMNs, stronger FASN expression correlated with higher histologic grade and the presence of an associated invasive carcinoma, whereas in PDAC, it correlated with vascular invasion (39). Additional clinicopathological studies linked FASN expression to tumor differentiation and potentially lymph-node metastasis (40), while high FASN expression was associated with poorer survival in patients with PDAC (27, 29). Experimental evidence further showed that FASN promotes PDAC cell proliferation and tumor growth (27), and its expression increases during PDAC progression in genetically engineered mouse models (29). Beyond its tissue expression, circulating FASN protein levels were also elevated in patients with PDAC and IPMN, suggesting potential utility as a circulating biomarker; however, its diagnostic and prognostic value remains to be established (39).

FASN upregulation in PDAC is sustained, at least in part, by oncogenic signaling. Epidermal growth factor receptor (EGFR) activation promotes FASN expression through the extracellular signal-regulated kinase (ERK) pathway, establishing an EGFR–ERK–FASN regulatory axis that links growth-factor signaling to *de novo* lipogenesis in PDAC. Consistent with this regulatory relationship, inhibition of EGFR or ERK reduces FASN expression, while disruption of this axis suppresses PDAC cell proliferation and tumor growth (27).

Beyond supporting tumor growth, FASN-dependent metabolic reprogramming contributes to therapeutic resistance through multiple mechanisms. FASN regulates pyruvate kinase M2 (PKM2) expression and glucose metabolism, thereby promoting gemcitabine resistance; conversely, FASN depletion decreases PKM2 expression and restores gemcitabine sensitivity, whereas PKM2 overexpression partially reverses this effect (41). FASN-dependent lipogenesis also supports gemcitabine resistance by maintaining tumor cell stemness and limiting ER stress. Accordingly, pharmacological inhibition of FASN synergizes with gemcitabine, increases ER stress and apoptosis, and reduces stemness in PDAC models (29). Independent studies further demonstrated that FASN overexpression increases resistance to both gemcitabine and irradiation, whereas genetic silencing or pharmacological inhibition of FASN enhances treatment-induced cell death (28).

Collectively, these findings suggest that FASN inhibition may offer the greatest benefit when combined with other modalities to overcome resistance rather than used alone. Its clinical relevance will depend on identifying tumors that are sustainedly dependent on *de novo* lipogenesis.

##### 2.1.1.2 Acetyl-CoA carboxylase: ACC

ACC is a key enzyme in FA metabolism that catalyzes the ATP-dependent conversion of acetyl-CoA to malonyl-CoA. Mammals express two ACC isoforms, ACC1 and ACC2, whose distinct subcellular localization functionally partitions malonyl-CoA between lipid synthesis and fatty acid oxidation (FAO) (42). ACC1 is predominantly cytosolic, where its proximity to the cytosolic lipogenic machinery allows the generated malonyl-CoA to serve as a substrate for FASN-dependent *de novo* FA synthesis, supporting the production of membrane and storage lipids (43). By contrast, ACC2 contains a unique N-terminal targeting sequence that anchors it to the outer mitochondrial membrane, where locally generated malonyl-CoA inhibits the adjacent carnitine palmitoyltransferase 1 (CPT1) and thereby restricts mitochondrial import and β-oxidation of long-chain FAs (44, 45). Thus, despite catalyzing the same biochemical reaction, the spatial segregation of ACC1 and ACC2 directs their malonyl-CoA products toward distinct metabolic fates: ACC1 primarily supports cytosolic lipogenesis, whereas ACC2 primarily regulates mitochondrial FAO.

This spatial compartmentalization also imposes distinct kinetic constraints. Cytosolic malonyl-CoA supports FASN-dependent lipogenesis, whereas malonyl-CoA generated near mitochondria inhibits CPT1 and thereby limits FAO (46, 47). CPT1 sensitivity to malonyl-CoA is further modulated by mitochondrial morphology, with fragmentation promoting FAO and elongation suppressing it (48). Thus, ACC1 inhibition mainly restricts lipogenesis, whereas ACC2 inhibition may enhance FAO by relieving CPT1 inhibition, providing a potential route for metabolic compensation.

In PDAC models, ACC inhibition reduced WNT3A lipidation and secretion, attenuated WNT/β-catenin and Hedgehog signaling, and suppressed PDAC cell proliferation (22). The ACC inhibitor BAY ACC002 further induced marked tumor regression in a gemcitabine-resistant xenograft model, suggesting that ACC blockade may retain antitumor activity in tumors with limited gemcitabine sensitivity (22). ACC activity can also be indirectly suppressed through upstream metabolic signaling; the lipoate analog CPI-613 activated AMP-activated protein kinase (AMPK) and increased inhibitory ACC phosphorylation, thereby reducing lipid accumulation and enhancing reactive oxygen species (ROS)-associated apoptosis in PDAC cells (49). However, ACC1 expression varies across PDAC cell lines, with relatively low levels reported in PANC-1 cells (50), suggesting heterogeneity in ACC-dependent lipogenesis. PANC-1 cells are known to be KRAS-mutant and exhibit mesenchymal features (51). Although metabolite profiling classified them as lipogenic, their transcriptional and drug-response profiles were more consistent with glycolytic cells (52). Sterol regulatory element-binding protein 1 (SREBP1) depletion reduces ACC expression in PDAC models, suggesting that variation in lipogenic transcriptional programs may contribute to this heterogeneity (53). However, the basis of low ACC1 expression in PANC-1 remains unclear.

Pharmacological ACC inhibition has shown antitumor activity in preclinical models of non-small-cell lung cancer and hepatocellular carcinoma (54, 55). Although ACC inhibitors have not yet been clinically established as anticancer agents, compounds such as firsocostat have advanced to Phase II clinical evaluation in metabolic liver disease (56), demonstrating the feasibility of pharmacological ACC inhibition in humans. Collectively, these findings support ACC as a potentially druggable metabolic vulnerability in PDAC, although its therapeutic efficacy is likely to depend on tumor-specific metabolic context.

#### 2.1.2 Extracellular fatty acid acquisition and nutrient scavenging

##### 2.1.2.1 CD36 and FATPs in exogenous fatty acid uptake

Uptake of exogenous FAs provides PDAC cells with an additional lipid source beyond *de novo* synthesis. FA uptake is conducted through membrane-associated transport proteins, including CD36 and FATP (57). CD36, known as a scavenger receptor class B type 2 (SR-B2), promotes FA uptake through regulated endocytosis. And inhibition of CD36 internalization markedly reduces CD36-dependent FA uptake (58). Some studies have shown that CD36-mediated FA uptake has been linked to metastasis and tumor growth, partly by supplying substrates for β-oxidation (59, 60).

In PDAC, CD36 can regulate chemoresistance. High CD36 expression correlates with gemcitabine resistance and poor outcomes, potentially through enhanced anti-apoptotic signaling (30). Consistently, gemcitabine plus nab-paclitaxel treatment increased CD36 expression in PDAC, while organoids derived from treated tumors showed enhanced exogenous oleic acid uptake (32). Oleic acid promoted PDAC cell growth and chemoresistance, whereas CD36 blockade or knockdown attenuated these effects, supporting a role for the CD36–oleic acid axis in chemotherapy adaptation (32).

In addition to CD36, the FATP family also contributes to exogenous FA acquisition. The FATP family comprises six members encoded by *SLC27A1–SLC27A6* and facilitates the uptake and metabolic activation of long-chain FAs (31). FATP-dependent lipid uptake enables tumor cells to exploit FAs supplied by the microenvironment. In PDAC, coculture with mature adipocytes increased FATP1 expression in tumor cells, accompanied by lipid accumulation, enhanced FAO, and increased invasive capacity, suggesting that FATP1 may contribute to the acquisition and utilization of adipocyte-derived lipids (61). *SLC27A2, SLC27A3, and SLC27A4* were upregulated in PDAC tissues compared with normal pancreatic tissues, with elevated *SLC27A3* expression additionally associated with lymph node involvement (62).

More recently, SMAD4 was shown to bind to the *SLC27A3* promoter and regulate its expression. SMAD4 deficiency reduced *SLC27A3* expression and decreased the abundance of linoleic acid, arachidonic acid (AA), and their downstream metabolites (63). This reduction limited the polyunsaturated FA pool available for lipid peroxidation and may contribute to resistance to radiotherapy-induced ferroptosis (63). Collectively, these findings suggest that the FATP family may represent another important route for the acquisition and utilization of exogenous FAs in PDAC, although the specific functions and direct metabolic roles of individual FATPs require further validation.

##### 2.1.2.2 KRAS-driven macropinocytosis in fatty acid uptake

Macropinocytosis is a non-selective, actin-dependent endocytic process. During this process, plasma membrane ruffles engulf extracellular fluid together with dissolved macromolecules and other extracellular materials, generating large intracellular vesicles known as macropinosomes (64). Macropinocytosis enables internalization of bulk extracellular materials to the endolysosomal system for degradation and metabolic reuse (64). This pathway is particularly relevant to PDAC, where oncogenic KRAS drives constitutive macropinocytosis and thereby increases extracellular nutrient uptake (65). Mechanistically, KRAS signaling regulates the localization and recycling of syndecan 1 (SDC1), which is an important mediator of macropinocytosis through the mitogen-activated protein kinase (MAPK)–PSD4–ARF6 axis, thereby maintaining the macropinocytic phenotype of KRAS-driven PDAC cells (65). Moreover, phosphoinositide 3-kinase (PI3K) signaling and the small GTPase RAC1 also participate in the membrane remodeling required for macropinosome formation (66).

Extracellular proteins are the most common substrates of macropinocytosis, particularly albumin. These proteins are internalized through macropinocytosis and subsequently delivered to lysosomes for proteolytic degradation (64, 67). The resulting amino acids replenish intracellular amino acid pools and support central carbon metabolism and macromolecule synthesis. This reduces the dependence of PDAC cells on freely available extracellular amino acids (68). PDAC tumors can actively catabolize extracellular proteins *in vivo*, whereas inhibition of macropinocytosis reduces intratumoral amino acid availability (67).

Macropinocytosis may also contribute to lipid acquisition in PDAC. Exogenous FAs such as oleic acid can be acquired through a macropinocytosis-dependent process in PDAC cells (33). And pharmacological inhibition of macropinocytosis or genetic suppression of RAC1 diminishes the protective effects conferred by extracellular FAs (33). Oncogenic RAS can also enhance scavenging of serum-derived unsaturated FAs supplied by lysophospholipids, thereby reducing dependence on *de novo* FA synthesis and desaturation (36). Together, these findings suggest that KRAS-driven macropinocytosis may help sustain lipid availability and metabolic flexibility in PDAC.

#### 2.1.3 Fatty acid desaturation and lipid homeostasis

FA desaturation helps maintain the balance between saturated and monounsaturated fatty acids (MUFAs), thereby supporting membrane composition and limiting lipid stress. SCD1 is a Δ9 desaturating enzyme located in the ER. By introducing a double bond into palmitoyl-CoA and stearoyl-CoA, stearoyl-CoA desaturase (SCD) generates palmitoleoyl-CoA and oleoyl-CoA, respectively, thereby promoting the conversion of saturated fatty acyl-CoAs into MUFAs. Pharmacological inhibition of SCD1 disrupts the balance between saturated and monounsaturated FAs, activates the unfolded protein response, and inhibits the growth of PDAC organoids and xenografts (69). This reaction represents a key desaturation step downstream of *de novo* FA synthesis, because the resulting MUFAs serve as major substrates for the synthesis of PLs, TAGs, and CEs (70, 71). By regulating the cellular saturated fatty acid (SFA)/MUFA ratio, SCD activity helps maintain membrane lipid composition and promotes the incorporation of FAs into neutral-lipid stores, thereby limiting SFA-associated lipotoxic stress (70, 72).

Among the five mammalian *SCD* genes (*SCD1–5*), humans express SCD1 and SCD5, with SCD1 serving as the predominant isoform in lipid metabolism and cancer biology (73). SCD1 is elevated in subsets of PDAC tissues and cell lines, and SCD1-dependent desaturation contributes to lipid homeostasis in PDAC cells (69, 74). Increasing evidence also links SCD1-mediated MUFA synthesis to ferroptosis resistance in PDAC. FBW7 suppresses SCD1 expression by reducing NR4A1 abundance and its binding to the *SCD1* promoter, whereas ZNF488 directly binds the *SCD1* promoter and enhances its transcription (34, 35). These reciprocal mechanisms modulate MUFA production and ferroptotic susceptibility. In addition, SLC25A22 promotes SCD1 expression and MUFA production through AMPK-dependent signaling in PDAC cells. Genetic or pharmacological inhibition of SCD1 sensitized these cells to RSL3-induced ferroptosis, whereas oleic acid supplementation improved cell survival after RSL3 or erastin treatment (75). Under nutrient deprivation, PANC-1 and PaTu8988T cells activated the mTOR–SREBP1–SCD1 axis and increased intracellular MUFA levels, thereby limiting lipid peroxidation and ferroptotic cell death (76). Depletion of SREBP1 or SCD1 restored ferroptosis sensitivity, while oleic acid reversed the increase in lipid ROS caused by SCD1 loss (76).

SCD1 also integrates mechanical and therapeutic signals from the TME. Increased matrix stiffness elevates SCD1 expression through Piezo1-mediated Ca²^+^ influx and PI3K–AKT signaling, promoting MUFA synthesis, TAG and LD accumulation, and gemcitabine resistance (77). Pharmacological or genetic inhibition of SCD1 reduced lipid accumulation and restored gemcitabine sensitivity, whereas exogenous oleic acid partially rescued the resistant phenotype (77). Together, these findings position SCD1 as a stress-responsive metabolic node through which transcriptional and microenvironmental signals reshape FA desaturation to support PDAC survival and therapeutic resistance (**Figure 1**).

**Figure 1:**
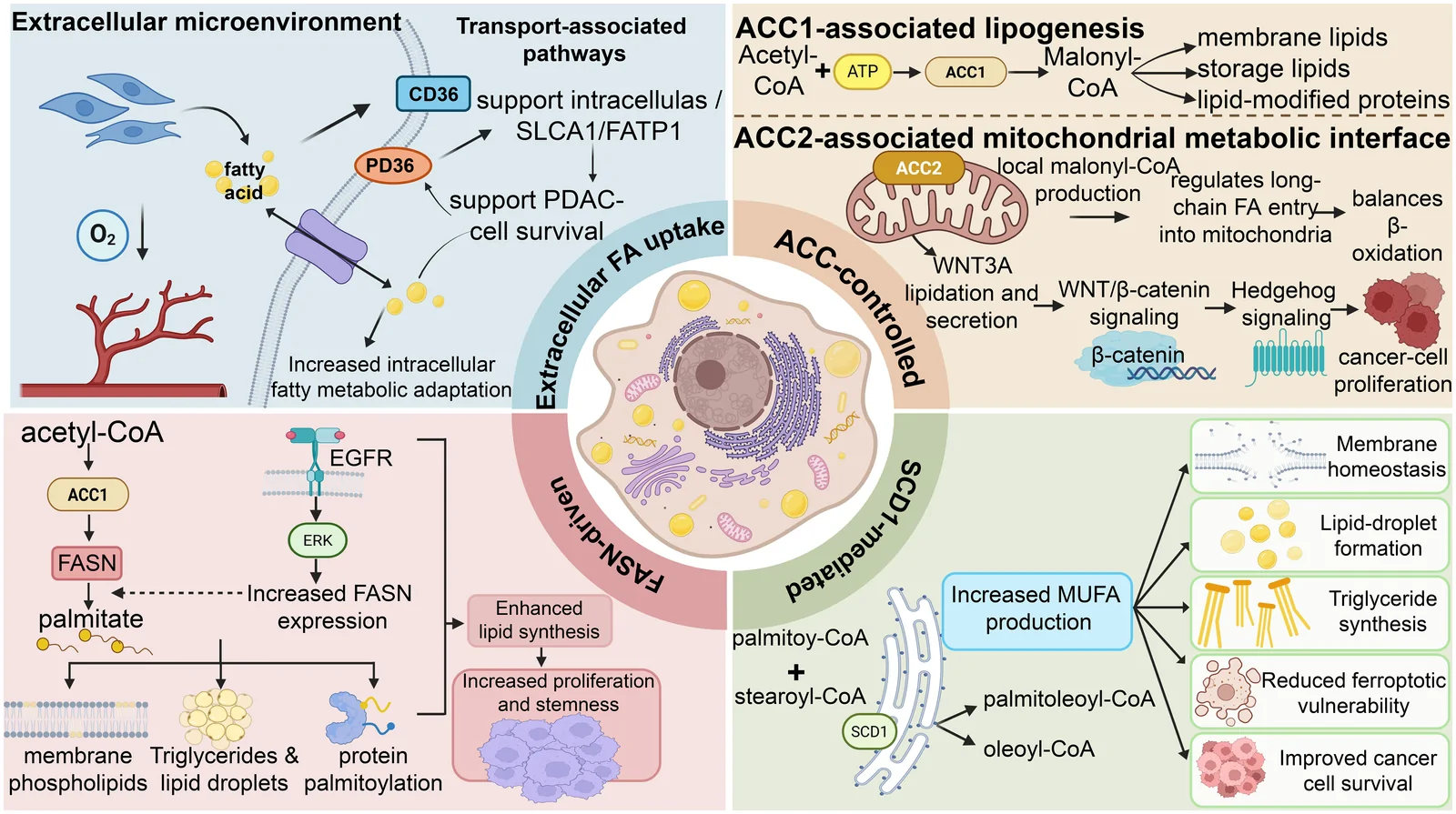
Fatty acid (FA) metabolic reprogramming in pancreatic ductal adenocarcinoma (PDAC). PDAC cells maintain FA homeostasis through interconnected programs of extracellular FA uptake, *de novo* lipogenesis, and FA desaturation. Extracellular FAs are acquired through transporter-associated pathways involving CD36, a scavenger receptor that facilitates FA uptake, and members of solute carrier family 27 (SLC27), also known as fatty acid transport proteins (FATPs). Acetyl-CoA carboxylase 1 (ACC1) and fatty acid synthase (FASN) drive *de novo* lipogenesis, which supplies FAs for membrane phospholipid synthesis, lipid storage, and protein lipidation. Acetyl-CoA carboxylase 2 (ACC2), by contrast, regulates mitochondrial fatty acid oxidation through malonyl-CoA production. Stearoyl-CoA desaturase 1 (SCD1)-mediated desaturation increases the production of monounsaturated FAs, thereby supporting membrane homeostasis, lipid storage, ferroptosis resistance, and tumor cell survival. Together, these pathways enable PDAC cells to adaptively regulate FA availability and composition in response to metabolic and microenvironmental stress.

### 2.2 Cholesterol and mevalonate metabolism

Cholesterol and the mevalonate pathway contribute to several processes important for PDAC growth, including membrane formation, lipid raft maintenance, and protein prenylation. In PDAC cells, cholesterol homeostasis is maintained through many routes, involving *de novo* synthesis, esterification of free cholesterol, and extracellular cholesterol acquisition. Accordingly, this section is organized around three interconnected modules: *de novo* cholesterol and non-sterol isoprenoid synthesis, extracellular cholesterol acquisition and cholesterol esterification and storage. These modules enable PDAC cells to redistribute cholesterol flux in response to nutrient limitation or therapeutic inhibition (78).

#### 2.2.1 *De novo* cholesterol and isoprenoid synthesis

##### 2.2.1.1 3-Hydroxy-3-methylglutaryl-coenzyme A reductase: HMGCR

HMGCR is an ER membrane protein that catalyzes the conversion of HMG-CoA to mevalonate, the rate-limiting step of the mevalonate pathway. Mevalonate serves as a central precursor for both sterol and non-sterol biosynthesis. In the sterol branch, it ultimately contributes to cholesterol production, supporting membrane structure and other sterol-dependent cellular functions. In parallel, the non-sterol branch generates isoprenoid intermediates, including farnesyl pyrophosphate (FPP) and geranylgeranyl pyrophosphate (GGPP), which provide prenyl groups for the post-translational modification of small GTPases from the RAS, RHO, and RAB families (79). Prenylation promotes their association with cellular and organelle membranes, enabling proper localization and signaling activity. Through these sterol and non-sterol outputs, the HMGCR–mevalonate pathway contributes to cholesterol and membrane homeostasis, vesicular trafficking, cytoskeletal organization, and oncogenic signaling (79, 80).

HMGCR activity is tightly controlled at multiple levels: sterol regulatory element-binding protein 2 (SREBP2) promotes its transcription, AMPK inhibits its activity through phosphorylation, and excess sterols accelerate its ubiquitin–proteasome-dependent degradation (81). At the protein level, HMGCR comprises an N-terminal ER membrane domain and a cytosolic C-terminal catalytic domain. Sterols and selected mevalonate-derived intermediates promote insulin-induced gene (INSIG)-dependent ubiquitination and proteasomal degradation of HMGCR, whereas AMPK-mediated phosphorylation of human HMGCR at Ser872 acutely suppresses its catalytic activity (80, 82, 83). These complementary regulatory layers enable rapid adaptation of mevalonate-pathway flux to changes in cellular sterol abundance and energy status.

In PDAC, increased HMGCR activity and mevalonate pathway flux are likely driven by several interacting factors rather than by a single abnormal feedback mechanism. Oncogenic signaling, metabolic substrate availability, and transcriptional regulation can all contribute to this regulation. During KRAS-driven pancreatic tumorigenesis, acetyl-CoA metabolism is extensively remodeled. Depletion of ATP citrate lyase (ACLY) reduces the amount of acetyl-CoA available for the mevalonate pathway and, in turn, limits acinar-to-ductal metaplasia (ADM) and subsequent tumor development (84). These findings suggest that KRAS provides a metabolic basis for the production of both sterol and non-sterol isoprenoid products by increasing substrate availability. Therefore, this relationship should not be oversimplified as direct transcriptional upregulation of *HMGCR* by KRAS.

Meanwhile, oxysterol-binding protein-related protein 5 (ORP5) promotes SREBP2 activation and downstream cholesterol biosynthesis, with ORP5-high PDAC cells showing increased sensitivity to HMGCR inhibition (85). Recent studies have also identified two more direct transcriptional regulatory axes of HMGCR. TRIB1 binds to the DNA-binding domain of peroxisome proliferator-activated receptor γ (PPARγ) and relieves PPARγ-mediated repression of *HMGCR* transcription (86). HOXC9, in contrast, transcriptionally activates *ITGA10* and subsequently upregulates *HMGCR* through FAK–PI3K–CREB signaling (87). Taken together, these findings indicate that HMGCR activity in PDAC is determined by the coordinated effects of metabolic substrate supply and tumor-specific transcriptional networks.

The tumor-supporting effects of the mevalonate pathway in PDAC appear to depend largely on its non-sterol products rather than on cholesterol accumulation itself. *In vivo* CRISPR screening identified GGPP synthesis within the mevalonate pathway as an important metabolic dependency for orthotopic PDAC growth. Silencing either *HMGCR* or geranylgeranyl diphosphate synthase 1 (GGPS1) reduced tumor burden, whereas combined inhibition of HMGCR and sterol regulatory element-binding protein (SREBP) disrupted small GTPase prenylation and induced apoptosis (23). These findings directly connect HMGCR-mediated metabolic flux with the membrane localization and signaling functions of RAS, RHO, RAB, and related proteins.

The mevalonate pathway also supports coenzyme Q10 (CoQ10) biosynthesis, linking non-sterol isoprenoid metabolism to redox control (88, 89). Ferroptosis suppressor protein 1 (FSP1) reduces CoQ10 to ubiquinol to suppress lipid peroxidation and ferroptosis independently of glutathione peroxidase 4 (GPX4) (90, 91). Although the FSP1–CoQ10 axis has been implicated in ferroptosis regulation in pancreatic cancer, its direct dependence on HMGCR-driven mevalonate metabolism remains to be established in PDAC.

However, the clinical value of targeting HMGCR remains uncertain. Observational studies of statin exposure have assessed several outcomes, including pancreatic cancer risk, disease stage at diagnosis, postoperative recurrence, and survival (92–94). But these results have not been consistent. While some cohort studies have suggested potential protective effects (95, 96), several large prospective studies have found no clear association (97–100). These epidemiological observations should therefore be interpreted cautiously, as they do not establish that HMGCR inhibition itself is responsible for a causal antitumor effect. Thus, although preclinical evidence supports that statins may exert antitumor effects and enhance sensitivity to chemotherapy, their therapeutic efficacy in PDAC remains to be established in prospective interventional studies.

##### 2.2.1.2 Squalene epoxidase: SQLE

SQLE, also known as squalene monooxygenase, acts downstream of HMGCR in the sterol biosynthetic pathway. It catalyzes the conversion of squalene to 2, 3(S)-oxidosqualene and serves as an important rate-limiting enzyme in this branch of cholesterol synthesis, which is the first oxygen-dependent step of the pathway. SQLE is a FAD-dependent monooxygenase localized to the ER membrane. The C-terminal region of SQLE contains the catalytic machinery responsible for substrate recognition and epoxidation, while its N-terminal region is involved in sensing cellular cholesterol levels and controlling protein turnover. When cholesterol is depleted, activated SREBP2 enters the nucleus and increases *SQLE* transcription. In contrast, cholesterol accumulation promotes MARCH6-mediated ubiquitination and proteasomal degradation of SQLE via its N-terminal degron, thereby reducing flux through the sterol biosynthetic pathway (101–103). Unlike HMGCR, SQLE not only regulates the rate of cholesterol synthesis but also directly controls intracellular squalene accumulation. Therefore, its biological activity can affect both downstream sterol production and the accumulation of squalene.

In PDAC, elevated SQLE expression has been reported in tumor tissues and is associated with poorer survival as well as enhanced proliferative and invasive behavior (24, 104, 105). Its expression is controlled by sterol feedback, non-coding RNAs, and KRAS-related stress responses. The long non-coding RNA TTN-AS1 binds miR-133b and reduces the inhibition of SQLE by miR-133b. This increases SQLE expression and activates ERK- and nuclear factor κB (NF-κB)-related signaling (105). In addition, during KRAS-driven adaptation to oxidative stress, the establishment of antioxidant homeostasis and the restoration of MYC activity have both been linked to sustained SQLE expression (106).

SQLE supports PDAC growth through multiple distinct mechanisms. First, SQLE sustains *de novo* cholesterol synthesis and cholesterol-dependent lipid raft integrity, thereby facilitating Src activation and downstream PI3K–AKT signaling to support PDAC cell proliferation and survival (24). SQLE depletion disrupts this cholesterol–lipid raft–Src/PI3K–AKT axis, whereas cholesterol replenishment partially restores pathway activation (24). Second, the accumulation of squalene following SQLE inhibition may also have direct antitumor effects. Squalene binds to glucose-regulated protein 78 (GRP78) and activates PERK–eIF2α–ATF4–CHOP signaling, ER stress, and apoptosis (24). Terbinafine, an antifungal agent with SQLE-inhibitory activity, and NB-598, a potent and selective mammalian SQLE inhibitor, both induced this response, with NB-598 showing greater potency *in vitro* (24). More recent evidence shows that squalene can also bind to the transcription factor Sp1 and increase its abnormal binding to the *TFAP2E* promoter. This suppresses *TFAP2E* expression and reduces the expression of its downstream target PGC-1α, thereby impairing mitochondrial biogenesis and energy metabolism (106).

SQLE-dependent removal of squalene also influences the TME. In KPC models, SQLE silencing induces squalene accumulation and suppresses p65/NF-κB-dependent *CXCL1* transcription, thereby reducing the infiltration of immunosuppressive myeloid populations, including myeloid-derived suppressor cells (MDSCs) and tumor-associated macrophages (TAMs), while increasing the proportion of intratumoral CD8^+^ T cells (107). These findings suggest that SQLE can influence PDAC progression not only through tumor-cell-intrinsic cholesterol metabolism but also by shaping the immune-cell composition of the TME.

Beyond its effects on the immune microenvironment, SQLE has also been linked to tumor-intrinsic therapeutic response. The SQLE inhibitor terbinafine increases the *in vitro* sensitivity of PDAC cells to gemcitabine, cisplatin, and 5-fluorouracil (24). Higher SQLE expression has also been observed in radioresistant cells and in residual tumor cells after neoadjuvant therapy; however, these observations are based largely on expression profiling and exploratory single-cell analyses and therefore do not establish a direct causal role for SQLE in treatment resistance (108, 109). Similarly, icaritin simultaneously downregulates HMGCR and SQLE and broadly remodels multiple lipid classes, making it difficult to attribute its antitumor effects specifically to SQLE inhibition (110).

#### 2.2.2 Extracellular cholesterol acquisition

##### 2.2.2.1 LDLR-mediated cholesterol uptake

In addition to *de novo* synthesis, PDAC cells can acquire exogenous cholesterol through low-density lipoprotein receptor (LDLR)-mediated uptake of circulating low-density lipoprotein (LDL). LDL binding to LDLR initiates clathrin-dependent endocytosis, followed by lysosomal hydrolysis of LDL-derived CEs to release free cholesterol for redistribution to cellular membranes and other organelles (111–113).

In PDAC, LDLR expression and LDL-dependent cholesterol uptake are increased compared with normal pancreatic tissue, suggesting an enhanced reliance on extracellular cholesterol acquisition. Functionally, LDLR silencing reduces cholesterol uptake, alters intracellular cholesterol distribution, suppresses ERK1/2 survival signaling, and sensitizes PDAC cells to gemcitabine (114). Consistently, elevated LDLR expression has been observed in both primary and metastatic PDAC, further supporting LDLR-mediated cholesterol uptake as an important component of cholesterol homeostasis in PDAC.

##### 2.2.2.2 NPC1L1-mediated cholesterol acquisition

Niemann–Pick C1-like 1 (NPC1L1) provides an additional route for extracellular cholesterol acquisition. It binds extracellular cholesterol and facilitates its uptake through cholesterol-regulated trafficking and clathrin dependent endocytosis (115, 116). Recent evidence indicates that NPC1L1 is ectopically upregulated in PDAC and directly contributes to cholesterol uptake. Genetic deletion of *Npc1l1* reduced exogenous cholesterol uptake in Panc-02 and KPC cells, whereas NPC1L1 overexpression enhanced it (117). Moreover, NPC1L1 enabled tumor cells to compete with CD8^+^ T cells for cholesterol in the TME, while pharmacological inhibition with ezetimibe reduced tumor-cell cholesterol uptake and enhanced the antitumor effects of programmed cell death protein 1 (PD-1) blockade (117). These findings identify NPC1L1 as a functionally relevant pathway for extracellular cholesterol acquisition in pancreatic cancer.

##### 2.2.2.3 SREBP2-mediated coordination of cholesterol synthesis and uptake

Cholesterol synthesis and extracellular uptake are coordinated through the SREBP cleavage-activating protein (SCAP)–SREBP2 feedback pathway. When ER cholesterol levels fall, SCAP dissociates from INSIG and escorts SREBP2 to the Golgi, where SREBP2 is proteolytically activated. Nuclear SREBP2 then induces genes involved in both cholesterol synthesis and uptake, including *HMGCR* and *LDLR* (118, 119). This coordinated response allows cells to increase endogenous cholesterol production while simultaneously enhancing LDL-derived cholesterol acquisition when intracellular cholesterol availability is reduced.

##### 2.2.2.4 Temporal and spatial constraints on compensatory cholesterol uptake

Compensatory cholesterol uptake is subject to both temporal and spatial constraints. The SCAP–SREBP2 system responds to the accessible ER cholesterol pool, and cholesterol acquired through endolysosomal pathways requires time to reach the ER and restore feedback control (120, 121). This response may also vary with nutrient availability, as SCAP trafficking is influenced by glutamine status (122). Thus, increased LDLR or NPC1L1 expression does not necessarily translate into sufficient cholesterol uptake when extracellular substrate availability is limited.

At the tissue level, compensatory uptake is further constrained by lipoprotein delivery to the tumor interstitial space. In PDAC, vascular heterogeneity, poor perfusion, and the desmoplastic stroma can limit substrate delivery, while interstitial nutrient concentrations differ from those in plasma and vary across tumors (123–126). Therefore, the balance between *de novo* cholesterol synthesis and extracellular uptake is likely to be temporally delayed, spatially heterogeneous, and incomplete.

#### 2.2.3 Sterol O-acyltransferase -mediated cholesterol esterification and storage

Once acquired from extracellular sources or generated through *de novo* synthesis, cholesterol must be appropriately distributed, utilized, or stored to prevent the accumulation of cytotoxic free cholesterol. SOATs catalyze the transfer of a long-chain fatty acyl group from fatty acyl-CoA to free cholesterol, generating CEs, a storage form of cholesterol that can be sequestered in LDs to buffer excess free cholesterol (127). Among the two SOAT isoforms, SOAT1, historically referred to as acyl-CoA:cholesterol acyltransferase 1 (ACAT1), is widely expressed and primarily localized to the ER membrane. By promoting cholesterol esterification and storage, SOAT1 reduces the potentially toxic accumulation of free cholesterol within the ER and other membrane compartments. Beyond this buffering function, SOAT1 lowers the metabolically sensed free-cholesterol pool, which can relieve negative feedback on SREBP2 and thereby sustain mevalonate pathway activity (9, 128).

During PDAC progression, increased SOAT1 expression is accompanied by enhanced CE accumulation (129). Using organoid and mouse models, Oni and colleagues showed that SOAT1 converts free cholesterol into relatively inert CEs. This process prevents free cholesterol from accumulating to levels that would trigger feedback suppression, thereby maintaining sustained expression of SREBP2, HMGCR, and other genes in the mevalonate pathway (128, 129). Genetic deletion or pharmacological inhibition of SOAT1 reduces CE storage, promotes free cholesterol accumulation, and induces ER stress, ultimately suppressing PDAC cell proliferation, orthotopic tumor growth, and metastasis. SOAT1 should therefore not be viewed merely as an enzyme involved in LD storage (129). Instead, cholesterol esterification allows it to couple the buffering of free cholesterol toxicity with persistent activation of the mevalonate pathway.

This metabolic dependency appears to be associated with a relatively specific genetic context. Loss of SOAT1 has a stronger inhibitory effect in PDAC organoids carrying mutant p53 together with *TP53* loss of heterozygosity (LOH). By comparison, organoids with wild-type p53, without *TP53* LOH, or with complete loss of p53 show a diminished response (128). This pattern indicates that SOAT1 dependence is associated with a specific *p53* genetic context rather than with TP53 alteration in general. Recent evidence further indicates that *TP53*-mutant PDAC exhibits higher SOAT1 expression and greater sensitivity to SOAT1 inhibition, suggesting that p53 status may help identify tumors with increased SOAT1 dependency, although its value as a predictive biomarker requires prospective clinical validation (130). Avasimibe, a pharmacological inhibitor of SOAT1, reduces CE formation and suppresses tumor growth in multiple PDAC models (129, 131). Avasimibe also sensitizes gemcitabine-resistant PDAC cells to gemcitabine, an effect associated with reduced CE accumulation and suppression of Akt signaling (132). However, these therapeutic findings are derived predominantly from cell-based, organoid, and animal models, and whether SOAT1 inhibition provides clinical benefit in molecularly selected patients with PDAC remains to be established (**Figure 2**).

**Figure 2:**
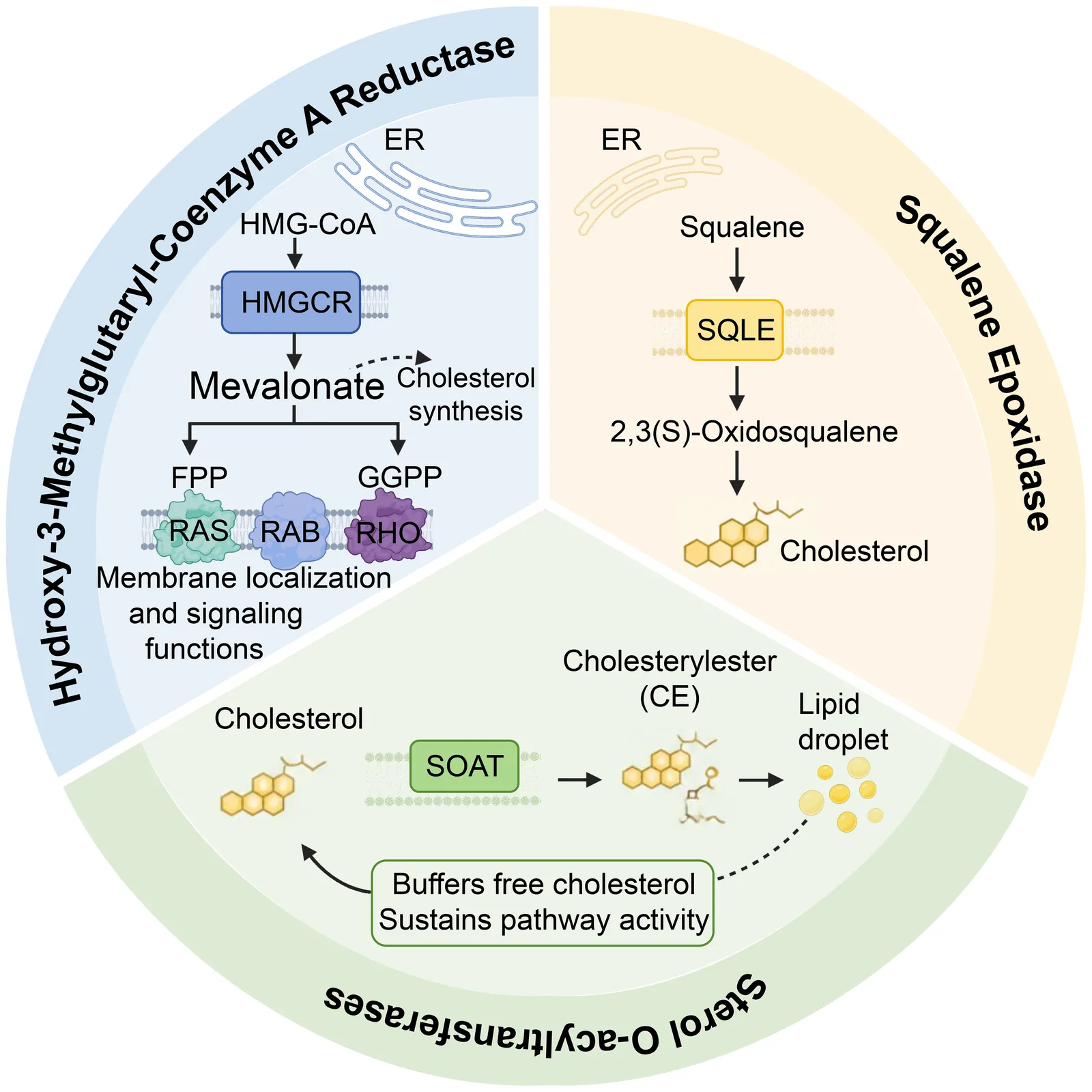
Cholesterol and mevalonate metabolism in pancreatic ductal adenocarcinoma (PDAC). PDAC cells maintain cholesterol homeostasis through interconnected pathways governing mevalonate production, sterol synthesis, and cholesterol esterification. 3-Hydroxy-3-methylglutaryl-CoA reductase (HMGCR) catalyzes the rate-limiting step of the mevalonate pathway, which generates sterol precursors and non-sterol isoprenoids, including farnesyl pyrophosphate (FPP) and geranylgeranyl pyrophosphate (GGPP). These intermediates support protein prenylation and thereby facilitate the membrane localization and signaling activity of small GTPases. Squalene epoxidase (SQLE) controls a key step in cholesterol biosynthesis by converting squalene into downstream sterol intermediates. Sterol O-acyltransferase 1 (SOAT1) esterifies free cholesterol to form cholesteryl esters, which are stored in lipid droplets, thereby buffering excess free cholesterol. Together, these pathways coordinate cholesterol synthesis, isoprenoid production, and cholesterol storage to maintain lipid homeostasis and support PDAC progression.

### 2.3 Lipid droplet dynamics: storage, lipolysis, and lipophagy

LDs are dynamic organelles that connect lipid storage with subsequent lipid mobilization. Excess FAs and cholesterol can be stored in LDs as neutral lipids, predominantly TAGs and CEs, thereby buffering fluctuations in lipid availability and limiting lipotoxic stress (9, 33). These stored lipids can subsequently be remobilized through cytosolic lipolysis or lysosomal lipophagy when metabolic demand increases (133). In PDAC, LD accumulation and turnover have been linked to tumor growth, invasion, and adaptation to therapeutic stress (16, 17, 134). Therefore, LDs in PDAC are not merely the structures of lipid storage but participate in diverse biological processes through the dynamic storage and mobilization of lipids.

#### 2.3.1 Lipid storage

In PDAC, lipid storage within LDs is actively regulated and can support malignant progression. HILPDA promotes TAG accumulation and LD formation in murine PDAC cells, whereas HILPDA depletion markedly reduces intracellular TAG storage and LD abundance and suppresses tumor growth *in vivo* (134). Nutrient availability can further determine whether FAs are oxidized or redirected into LDs. Branched-chain amino acid deprivation reduces mitochondrial FA import in PDAC and redirects FAs toward TAG synthesis and LD accumulation, an effect that is attenuated by DGAT1 inhibition (135).

LD accumulation is also increased in pancreatic cancer stem cells (CSCs), where it is accompanied by peroxisome proliferator-activated receptor α (PPARα) activation. Pharmacological or genetic inhibition of PPARα, as well as disruption of lipid re-esterification or lipolysis, impairs CSC properties (136). In addition, LD accumulation has been linked to therapeutic resistance. LPCAT2 is upregulated in pancreatic cancer and promotes LD formation, whereas LPCAT2 depletion or inhibition of LD accumulation reduces cell motility and sensitizes pancreatic cancer cells to gemcitabine (17). Together, these findings indicate that LD storage in PDAC provides more than a passive depot for excess lipids; it supports metabolic flexibility, malignant phenotypes, and adaptation to therapeutic stress.

#### 2.3.2 Neutral lipolysis

Neutral lipolysis mobilizes TAG stored in LDs through sequential hydrolysis by cytosolic lipases. Adipose triglyceride lipase (ATGL)/PNPLA2 initiates TAG hydrolysis, followed by hormone-sensitive lipase (HSL) and monoacylglycerol lipase, thereby releasing FAs for subsequent metabolic use (133). This pathway is closely linked to oncogenic KRAS signaling in PDAC. KRAS suppresses HSL expression through mitogen-activated protein kinase kinase (MEK)/ERK signaling, leading cells toward LD accumulation and lipid storage (16). However, these lipid reserves are subsequently required during invasive growth. Pharmacological inhibition of ATGL or HSL reduces PDAC cell invasion, whereas LDs are progressively consumed during invasive migration together with increased oxidative metabolism (16). Thus, KRAS appears to coordinate lipid storage with subsequent lipolytic mobilization, enabling PDAC cells to preserve lipid reserves that can later support invasion and metastasis.

#### 2.3.3 Lipophagy

In addition to cytosolic lipolysis, LDs can be mobilized through lipophagy, a selective autophagic process that delivers LDs to lysosomes for degradation (137). Although poorly studied, lipophagy appears to play a dual role in cancer. Under nutrient deprivation, enhanced lipophagic degradation of LDs supports the viability of hepatocellular carcinoma cells (138). Conversely, lipophagy may also exert tumor-suppressive effects in certain settings. For example, ATG14 overexpression enhances lipophagic LD degradation and increases the sensitivity of HeLa cells to apoptosis (139).

Lipophagy is elevated in human PDAC and has been associated with poor prognosis (140). OSBPL10 could promote lipophagy and tumor growth through VAPA/VAPB and ATG2A-mediated lysosomal repair, thereby facilitating the mobilization of lipids stored in LDs (140). Under metabolic stress, intracellular lipid recycling may become increasingly important. Restriction of extracellular lipid supply increases autophagic flux in PDAC, suggesting a compensatory response when exogenous lipids are limited (141). When glycolysis is suppressed, PDAC becomes increasingly dependent on mitochondrial FA metabolism to sustain energy production and cell survival. Impairment of lipophagy reduces LD–lysosome contact and leads to LD accumulation, thereby impairing this FA-dependent metabolic adaptation (142). Lipophagy also becomes important during therapeutic suppression of KRAS signaling. KRAS, MEK, or ERK inhibition activates transcription factor EB and increases lipophagy-dependent lipid turnover and FAO in PDAC, thereby supporting tumor cell survival (18) (**Figure 3**).

**Figure 3:**
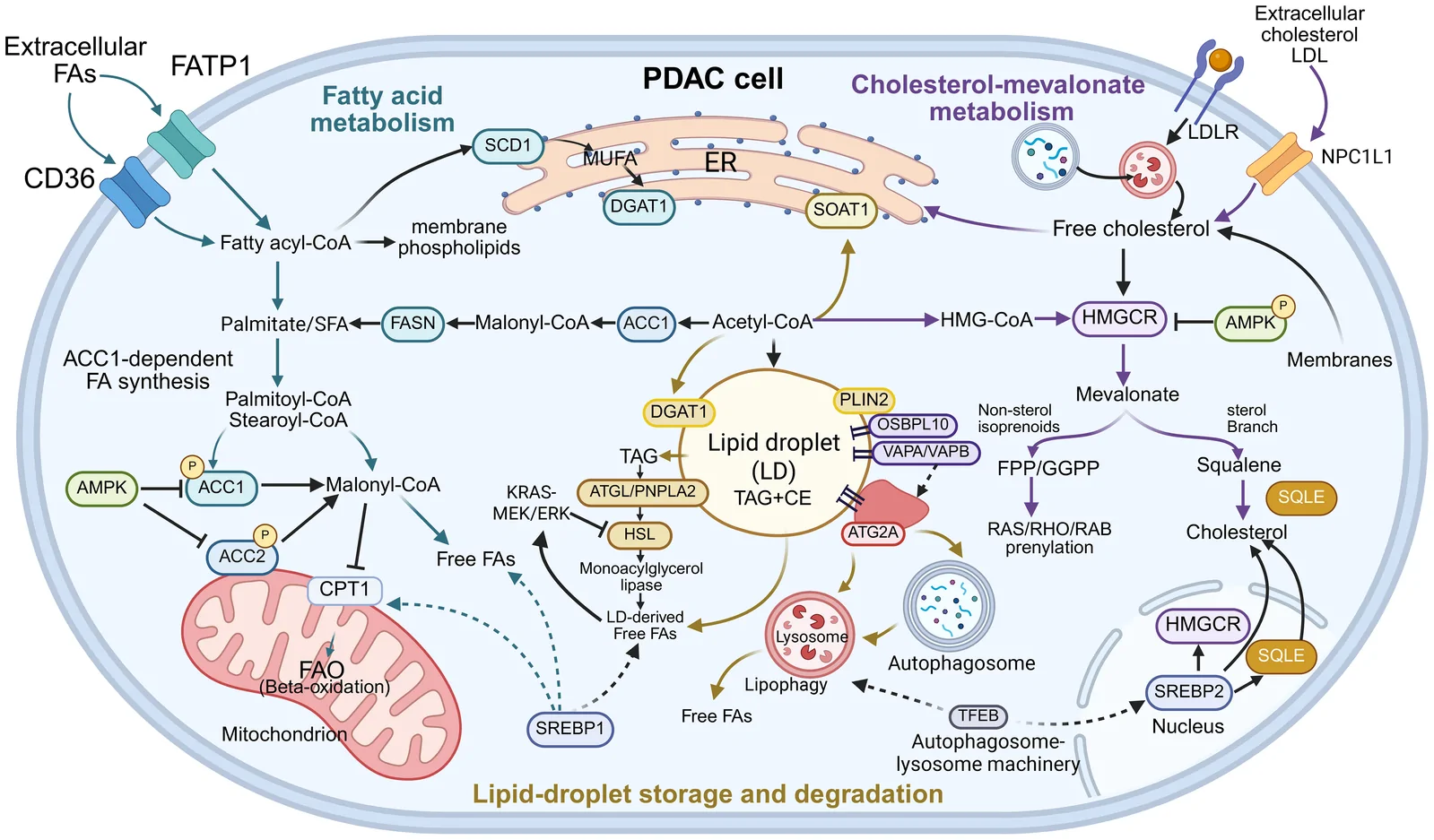
Integrated tumor-intrinsic lipid metabolic pathways in pancreatic ductal adenocarcinoma (PDAC). Citrate-derived acetyl-CoA provides a central metabolic link between fatty acid (FA) synthesis and cholesterol–mevalonate metabolism. Acetyl-CoA carboxylase (ACC) and fatty acid synthase (FASN) drive *de novo* FA synthesis, whereas stearoyl-CoA desaturase 1 (SCD1) regulates FA desaturation and lipid composition. In parallel, 3-hydroxy-3-methylglutaryl-CoA reductase (HMGCR) directs acetyl-CoA-derived carbon into the mevalonate pathway, supporting cholesterol synthesis and non-sterol isoprenoid production. Excess FAs and cholesterol can be incorporated into triglycerides and cholesteryl esters and stored in lipid droplets (LDs), which can subsequently be mobilized through lipolysis or lipophagy under metabolic stress. These interconnected pathways influence membrane homeostasis, endoplasmic reticulum (ER) stress, redox balance, and ferroptosis susceptibility. Together, the integrated network illustrates how PDAC cells coordinate lipid synthesis, desaturation, storage, and recycling to maintain lipid homeostasis and adapt to metabolic stress.

## 3 Lipid metabolic reprogramming in the PDAC tumor microenvironment

Lipid metabolic reprogramming in PDAC is influenced not only by malignant cells but is also shaped by the surrounding TME. The dense and poorly perfused stroma creates hypoxia, nutrient limitation, and metabolic competition, forcing both tumor and non-malignant cells to adapt their use of available lipids (143, 144). Within this environment, cancer-associated fibroblasts (CAFs), immune cells, adipocytes, and neural-associated components can influence lipid uptake, storage, release, utilization, and lipid-mediated signaling, while PDAC cells can, in turn, reshape the metabolic state of these surrounding populations (145–147). Through this bidirectional communication, lipids serve not only as metabolic and structural substrates but also as signaling mediators that influence stromal remodeling, immune suppression, tumor invasion, and treatment response. The following sections therefore review how lipid metabolism contributes to the functional interactions between PDAC cells and major cellular components of the TME (**Figure 4**).

**Figure 4:**
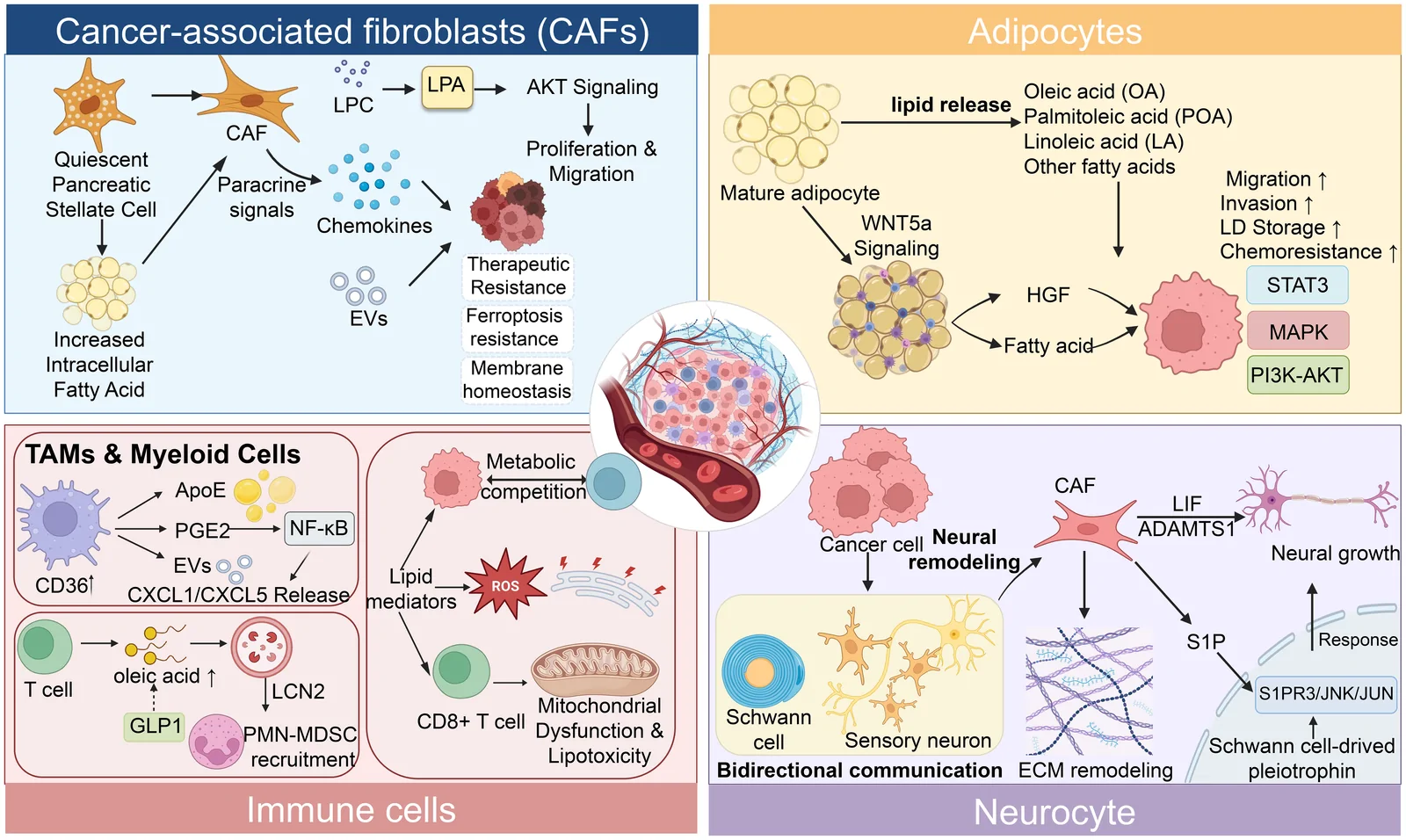
Lipid metabolic interactions between pancreatic ductal adenocarcinoma (PDAC) cells and the tumor microenvironment (TME). Cancer-associated fibroblasts (CAFs), adipocytes, immune cells, and neural-associated cells engage in lipid-dependent metabolic and signaling interactions with PDAC cells. CAFs regulate lipid availability and lysolipid signaling through fatty acids (FAs), the lysophosphatidylcholine–lysophosphatidic acid axis, paracrine factors, and extracellular vesicles. Adipocytes provide exogenous FAs and activate signaling programs associated with lipid storage, invasion, and chemoresistance. Lipid-related pathways in tumor-associated macrophages, myeloid cells, and T cells contribute to metabolic competition, inflammatory signaling, and immune dysfunction. Neural-associated interactions further connect lipid-derived mediators with extracellular matrix remodeling, Schwann-cell responses, and neural growth. Together, these cell-specific programs illustrate how lipid metabolism shapes stromal support, immune regulation, and neural remodeling in the PDAC microenvironment.

### 3.1 Cancer-associated fibroblasts

CAFs are major stromal components in PDAC, often comprising a substantial portion of the tumor mass and contributing to the dense desmoplastic microenvironment (148). CAFs influence tumor initiation and progression through multiple mechanisms, including paracrine secretion of chemokines, extracellular vesicles (EVs), metabolite exchange, and extracellular matrix (ECM) remodeling (149, 150). On the one hand, as a hallmark of PDAC, the desmoplastic stroma supports tumor invasion, metastasis, recurrence, therapeutic resistance, and immune evasion; on the other hand, excessive matrix deposition compresses blood vessels and impairs tissue perfusion, thereby aggravating intratumoral hypoxia and nutrient limitation (143). To achieve the energy requirements under these metabolically restrictive conditions, PDAC cells reprogram multiple metabolic pathways, including glucose, amino acid, glutamine, and lipid metabolism (144, 151). However, this adaptation is not entirely tumor-cell autonomous. Rather, CAFs also undergo metabolic reprogramming and support tumor growth through the reciprocal exchange of cytokines, chemokines, and metabolites, especially in lipid metabolism (21, 152). This bidirectional metabolic communication allows tumor cells to sustain biosynthesis, membrane homeostasis, and stress tolerance under otherwise unfavorable conditions.

#### 3.1.1 CAF heterogeneity and metabolic diversity

CAFs are not a homogeneous population, and their metabolic functions vary across distinct fibroblast states. Single-cell studies have identified several CAF states in PDAC, including the well-established myofibroblastic CAFs (myCAFs) and inflammatory CAFs (iCAFs), as well as the more recently described metabolic CAFs (meCAFs) (153). These meCAFs are characterized by enrichment of glycolytic and mitochondrial translation-related programs. Compared with myCAFs, meCAFs show greater expression of glycolytic enzymes such as LDHA and PKM2, whereas cancer cells in meCAF-rich tumors preferentially exhibit oxidative phosphorylation, suggesting potential metabolic coupling between these compartments (153). However, the original characterization of meCAFs did not directly identify the metabolites exchanged with tumor cells, and their specific role in lipid exchange remains incompletely defined.

#### 3.1.2 Lipid metabolic differences between myCAFs and iCAFs

Lipid metabolic states differ between canonical myCAFs and iCAFs. Single-cell metabolic analysis showed that iCAFs exhibit greater activity in FA metabolism, together with broader carbohydrate and amino acid metabolic activity, whereas myCAFs are relatively enriched in the tricarboxylic acid cycle and oxidative phosphorylation (154). Consistent with this distinction, direct lipidomic profiling showed that iCAF-like cells contain higher levels of multiple lipid classes, including ceramides (Cer), sphingomyelins (SM), phosphatidylethanolamines (PE), lysophosphatidylethanolamines (LPE), phosphatidylcholines (PC), lysophosphatidylcholine (LPC) and fatty acyl-CoAs (155). But when primary pancreatic stellate cells (PSCs) were experimentally differentiated into myCAFs or iCAFs, both populations produced comparable amounts of LPC (152). The two states also similarly induced autotaxin secretion from PDAC cells, indicating that LPC supply and downstream lysolipid signaling are not restricted to one canonical CAF phenotype (152).

#### 3.1.3 Lipid plasticity of PSCs

PSCs, an important cellular source of CAFs in PDAC, provide a well-characterized example of stromal lipid storage and plasticity (156, 157). In the quiescent state, PSCs store vitamin A within cytoplasmic LDs and show relatively greater lipid biosynthetic activity than other stromal cell types (157). During pancreatic tumorigenesis, PSCs differentiate into an activated fibroblastic state with profound lipid metabolic reprogramming, including reduced characteristic LDs and increased lipid secretion (152). Similarly, when treated with retinol or p53 activation, activated PSCs are driven back to a quiescent state, accompanied by increased levels of TAGs and CEs, indicating that stromal lipid reprogramming is not irreversible but rather a dynamic, reversible process (158, 159). The endogenous lipid mediator lipoxin A4 can similarly reprogram activated PSCs toward a quiescent-like phenotype, reduce collagen deposition, and suppress pancreatic tumor development (160). These observations indicate that lipid storage and mobilization are not merely passive markers of PSC activation, but rather dynamic, partially reversible processes that contribute to stromal plasticity in PDAC.

#### 3.1.4 CAF-derived lipid signaling

Beyond serving as markers of stromal cell state, lipids released from CAFs and PSCs can also be used directly by PDAC cells. Lipidomic analysis and isotope-tracing studies have demonstrated that FAs derived from PSCs are taken up by tumor cells and incorporated into PL and TAG pools, where they can contribute to membrane production and cellular energy metabolism (152). LPC is one of the stromal lipids readily taken up by PDAC cells, and it can be incorporated into PCs and contribute to membrane synthesis (21, 36). LPC also participates in signaling rather than functioning only as a metabolic substrate. After autotaxin-mediated conversion to lysophosphatidic acid, LPC-derived metabolites activate AKT signaling and promote PDAC cell proliferation and migration (152, 161). Thus, CAF-derived lipids provide both structural and metabolic substrates while simultaneously enhancing malignant phenotypes through lipid-mediated signaling pathways.

#### 3.1.5 CAF-mediated lipid supply

The dense stroma generated by CAFs aggravates tumor hypoxia to increase the reliance of PDAC cells on exogenous lipids (162). Oxygen limitation restricts unsaturated lipid synthesis, leading to the accumulation of saturated lipid species and increased ER stress. As a result, PDAC becomes more dependent on extracellular unsaturated lipids to maintain lipid balance, membrane fluidity, and the integrity of organelle membranes (36, 163). Recent evidence further suggests that this lipid-supporting capacity may vary according to CAF phenotype and spatial context. The CAFs used to demonstrate unsaturated LPC supply were predominantly myCAF-like, expressing α-smooth muscle actin and desmin (21). *In vivo*, myCAFs showed relatively low hypoxic stress and were positioned closer to functional vasculature, potentially allowing them to maintain unsaturated lipid production within the severely hypoxic PDAC microenvironment (21). These CAF-derived unsaturated LPCs are preferentially taken up by stressed PDAC cells and incorporated into PCs, thereby maintaining membrane integrity and suppressing activation of the IRE1α/XBP1 branch of the unfolded protein response (21). In contrast, human iCAFs showed a stronger hypoxic transcriptional signature, and their specific contribution to PDAC lipid homeostasis remains less clearly defined (21). These spatial differences therefore provide another potential layer of subtype-specific regulation of stromal lipid supply. However, this lipid-mediated protection is metabolically interconnected with other nutrient pathways. Blocking glutamine utilization can activate amino acid deprivation sensors and induce stress responses via ATF4/CHOP, thereby reversing the suppression of apoptosis by unsaturated LPC (164).

#### 3.1.6 CAF lipid transfer and resistance

CAFs may also contribute to therapeutic resistance through lipid-related mechanisms. CAF-derived EVs can suppress ferroptosis and promote gemcitabine resistance in PDAC cells by delivering miRNAs targeting acyl-CoA synthetase long-chain family member 4 (ACSL4), thereby impairing long-chain FA metabolism and reducing ferroptotic vulnerability (165). At the same time, cancer cells can reciprocally educate adjacent non-malignant cells to adopt tumor-promoting traits by changing their metabolite transfer (166, 167). In PDAC, tumor-intrinsic loss of *SETD2* promotes BMP2 signaling through the ectopic acquisition of H3K27ac, which drives fibroblasts toward an ABCA8a-positive, lipid-laden CAF state (146). These lipid-loaded CAFs can then transfer lipids to tumor cells through ABCA8a, supporting mitochondrial oxidative phosphorylation and tumor growth (146). This ABCA8a-positive lipid-laden population was identified independently of the previously described meCAF state and provides more direct evidence for a specialized lipid-donating CAF phenotype.

### 3.2 Immune cells

During tumor initiation and progression, malignant and immune cells engage in continuous and complex interactions (168). Tumor cells promote immune escape by diminishing antigen presentation and immunogenicity while simultaneously establishing an immunosuppressive microenvironment enriched with TAMs, regulatory T cells (Tregs), and MDSCs (169). These cells further suppress antitumor immunity by releasing inhibitory cytokines and other immunomodulatory factors (169). Because of insufficient vascularization and excessive ECM deposition, nutrient availability within the tumor is limited (170). This creates strong metabolic competition between cancer cells and immune cells, which can further impair immune cell function and weaken antitumor immunity (170). For example, immunosuppressive populations such as M2-like TAMs and Tregs commonly exploit lipid accumulation to maintain their functional programs. By contrast, CD8^+^ T cells and natural killer cells (NK cells) are particularly susceptible to lipid metabolic dysfunction under prolonged immune and metabolic stress, resulting in progressive loss of antitumor activity (171–173). Some lipid metabolic abnormalities, such as insufficient cholesterol overload and phospholipid synthesis, can act upstream of oxidative stress and ER stress, which in turn promote the canonical exhaustion of CD8^+^ T cells (174, 175). Conversely, CD36 in M2-like TAMs enhances the maintenance of a protumor phenotype by promoting FA uptake, storage, mitochondrial FAO, and oxidative phosphorylation (176, 177). Thus, lipid metabolism represents a critical metabolic interface between tumor immune evasion and microenvironmental immunosuppression.

#### 3.2.1 Lipid metabolism in T cells

##### 3.2.1.1 CD8^+^ T cell

FA metabolism is an important determinant of CD8^+^ T-cell dysfunction in the immunosuppressive PDAC microenvironment. Although immune cells are abundant in PDAC, CD8^+^ T-cell infiltration is frequently limited, and infiltrating CD8^+^ T cells often exhibit dysfunctional or exhausted phenotypes (178, 179). Lipid accumulation in the TME increases the long-chain FA burden in CD8^+^ T cells while reducing ACADVL/VLCAD-dependent FA catabolism, resulting in mitochondrial dysfunction, lipotoxic stress, and impaired T-cell persistence (145). Conversely, CD8^+^ T-cell lipid metabolism can itself reshape the surrounding immune microenvironment. Upon immune activation, infiltrating CD8^+^ T cells enhance FA synthesis, particularly oleic acid production, which induces lysosomal membrane permeabilization and subsequently activates NF-κB and promotes lipocalin 2 (LCN2) expression (180). Glucagon-like peptide 1 attenuates this process by inhibiting oleic acid synthesis, thereby reducing LCN2-mediated recruitment of polymorphonuclear myeloid-derived suppressor cells (PMN-MDSCs) and enhancing the efficacy of viral immunotherapy in PDAC (180). These observations suggest that restoring lipid-utilization capacity or limiting lipid-induced stress may be a potential strategy to preserve cytotoxic T-cell function in PDAC.

Cholesterol metabolism also shapes CD8^+^ T-cell immunity in PDAC through metabolic competition and tumor-cell antigen presentation. Ectopic NPC1L1 expression in PDAC enables tumor cells to overcome cholesterol shortages while sequestering intracellular cholesterol from CD8^+^ T cells, thereby impairing T-cell receptor activation and cytotoxic function (117). In addition, *cholesterol 25-hydroxylase (CH25H)* is epigenetically downregulated in PDAC, leading to enhanced autophagy-dependent major histocompatibility complex class I (MHC-I) degradation and limited CD8^+^ T cell infiltration (181). Restoration of *CH25H* expression can inhibit autophagy and recover MHC-I-dependent antigen presentation, which may improve the response of PDAC tumors to immune checkpoint blockade (181). Clinically, in patients with PDAC, higher levels of plasma total cholesterol and TAGs are also associated with lower CD8^+^ T cell infiltration and poorer clinical outcomes (182).

##### 3.2.1.2 CD4^+^ T cell

Compared with CD8^+^ T cells, the relationship between lipid metabolism and CD4^+^ T cell subsets has been less studied in PDAC. Treg cells could selectively upregulate CD36, which facilitates FA uptake, supports Tregs’ survival and immunosuppressive function (183). This enhanced capacity for free FA uptake and utilization allows Tregs to acquire a metabolic advantage, thereby promoting their accumulation and contributing to resistance to anti-PD-1 therapy (184). Lipid metabolism may also affect other CD4^+^ T cell subsets. In melanoma and colon adenocarcinoma models, inhibition of ACC1-dependent FA synthesis promotes T helper 9 (Th9) differentiation and enhances antitumor immunity (185). However, whether ACC1 inhibition similarly promotes Th9 differentiation and antitumor activity in PDAC remains unproven. The fibrotic stroma of PDAC may restrict T-cell infiltration (186), potentially limiting the therapeutic impact of enhanced Th9 differentiation.

#### 3.2.2 TAM

##### 3.2.2.1 TAM-mediated lipid signaling

TAMs are also closely related to lipid metabolic changes in the PDAC microenvironment. Apolipoprotein E released from TAMs can increase CXCL1 and CXCL5 expression in PDAC cells through LDLR and NF-κB signaling (187). These chemokines promote the recruitment of immunosuppressive myeloid cells and reduce CD8^+^ T-cell infiltration, further contributing to an immunosuppressive tumor environment (187). In obesity-associated PDAC TAMs, fatty acid-binding protein 4 (FABP4) facilitates saturated FA transfer and induces caspase-1/GSDMD-dependent pyroptosis (188). This activates the NLRP3/IL-1β inflammatory axis and promotes migration, invasion, and metastasis of PDAC cells (188). Lipid-derived inflammatory mediators also shape TAMs’ function. AA-derived prostaglandin E_2_ (PGE_2_), together with TNF, promotes the formation of IL-1β^+^ TAMs, which interact with inflammatory tumor-intrinsic IL-1β response signature^+^ tumor cells and maintain tumor-promoting inflammation (189). Blocking of PGE_2_ or IL-1β can alter the activity of TAMs and reduce inflammation, highlighting lipid mediator-driven macrophage inflammation as a therapeutically targetable vulnerability in PDAC (189). In addition, tumor-derived lactate increases lipid accumulation in TAMs and decreases IRF7 stability by inhibiting UFL1-mediated UFMylation, which favors protumoral macrophages (190).

##### 3.2.2.2 EV-mediated lipid metabolism

TAMs can also regulate tumor progression through lipid-related EV communication. Lipid-laden TAMs in the liver transfer lipids through exosomes, which are subsequently taken up by CD8^+^ T cells (191). The accumulation of macrophage-derived lipids impairs mitochondrial function and promotes CD8^+^ T cell exhaustion, thereby establishing an immunosuppressive hepatic microenvironment that facilitates liver metastasis of PDAC (191). Conversely, KRAS-G12D mutant proteins can be transferred from tumor cells to TAMs via exosomes, thereby enhancing signal transducer and activator of transcription 3 (STAT3)-dependent FAO, promoting TAM polarization characterized by IL10, ARG1, and TGFB1 expression, and ultimately driving PDAC progression (192). Notably, not all macrophage-associated lipid programs are tumor-promoting. Evidence from single-cell RNA sequencing and spatial transcriptomic analyses indicates that IRF7 overexpression in TAMs suppresses RPS18 transcription, reduces the exosomal delivery of RPS18 to tumor cells, and decreases ILF3 expression, along with lipid metabolite abundance (193). These changes are associated with reduced tumor-cell viability, proliferation, migration, invasion, and orthotopic tumor growth, while apoptosis is increased (193). TAM behavior in PDAC is influenced by several lipid-related processes, and through these mechanisms, macrophages can contribute to immune suppression, inflammatory remodeling, and tumor progression.

#### 3.2.3 Myeloid/MDSC

Beyond lymphocytes and macrophages, lipid metabolism also contributes to PDAC-associated immune suppression by reshaping other innate immune compartments. On the one hand, activated CD8^+^ T cells can promote PMN-MDSC recruitment through the oleic acid–LCN2 axis, thereby weakening antitumor immunity (180). On the other hand, during the progression from pancreatic intraepithelial neoplasia (PanIN) to PDAC in the KPC model, intratumoral polymorphonuclear neutrophils (PMNs) gradually shift from CD14^+^ PMNs toward CD14 ^int/high^ PMNs, with CD14 ^high^ PMNs becoming predominant at the PDAC stage (194). These CD14 ^high^ PMNs exhibit higher expression of lipid-related genes, including Slc27a2/FATP2 and Cd36, suggesting lipid metabolic alterations in suppressive neutrophil-like myeloid populations (194). In addition, lipid accumulation in dendritic cells can impair antigen cross-presentation, whereas lipid mediators such as PGE_2_ may disrupt NK cell-dependent type 1 conventional dendritic cells recruitment (195, 196). However, these findings remain to be further validated in the context of PDAC.

### 3.3 Adipocytes

In various solid tumors, adipocytes are no longer regarded merely as energy-storage or structural cells, but rather as active microenvironmental components that regulate tumor growth and progression. For example, in ovarian cancer, lipids released from omental adipocytes can be taken up and used by tumor cells, while loss of FABP4 markedly reduces metastatic growth (197). In breast cancer, adipocytes close to breast tumors can also release FAs and gradually lose their TAG stores, thereby acquiring a cancer-associated adipocyte (CAA) phenotype that supports tumor progression (198, 199). Although adipocytes are not the dominant stromal population in PDAC, their roles may become more important when pancreatic fat infiltration or tumor invasion occurs into surrounding adipose tissue.

#### 3.3.1 Adipocyte-derived lipid supply

The pancreas is a retroperitoneal organ surrounded by a large amount of adipose tissue, which may directly interact with tumor cells when PDAC invades beyond the pancreas. During extra-pancreatic invasion, PDAC cells directly encounter and remodel the peripancreatic adipose tissue, and this process promotes the release of FAs such as palmitoleic acid, oleic acid, and linoleic acid (147). These adipocyte-derived FAs increase LD formation and are also associated with increased invasion, migration, and gemcitabine resistance in PDAC (147). Co-culture with mature adipocytes further increases LD accumulation and FA β-oxidation in PDAC cells (61). At the same time, FATP1, DGAT1/2, GPAT4, and PLIN2 are upregulated, which may support FA uptake, TAG synthesis, lipid storage, and the use of stored lipids (61).

#### 3.3.2 CAA-mediated tumor–adipocyte crosstalk

The interaction between adipocytes and PDAC cells is not simply a unidirectional transfer of FAs. Besides receiving lipids from adipocytes, PDAC cells can alter nearby adipocytes and promote their transition into CAAs. In human PDAC specimens, adipocytes close to tumor cells are smaller than those in adjacent normal pancreatic tissue and exhibit impaired glucose and lipid metabolism (200). These adipocytes also express higher levels of fibroblast-related markers and may participate in stromal remodeling and tumor invasion (200). Mechanistically, WNT5a signaling has been reported to mediate the dedifferentiation of nearby adipocytes induced by PDAC cells (201). CAAs can also release chemokines, cytokines, and growth factors such as IL-6, IL-8, IL-10, MCP-1, TGF-β, M-CSF, VEGF, and HGF, which promote tumor growth, migration, and microenvironmental remodeling through MAPK, STAT3, and PI3K/AKT signaling (202, 203). Among these factors, adipocyte-derived HGF has been reported to promote PDAC cell proliferation (203). Therefore, the effects of CAAs are not limited to lipid supply, as their secreted factors can also influence tumor behavior. CAA-conditioned medium enhanced PDAC cell invasion, migration, chemoresistance, and epithelial–mesenchymal transition (EMT), and this effect was associated with increased serum amyloid A1 (SAA1) expression (204). Clinically, SAA1 positivity was detected in 46 of 61 human PDAC specimens and was associated with significantly shorter overall survival (204).

### 3.4 Neural-associated cells

Tumor-associated nerves are increasingly recognized as active components of the TME rather than merely structural substrates for cancer pain and perineural invasion (205, 206). Through interactions with malignant and stromal cells, neural-associated cells participate in tumor progression and microenvironmental remodeling. In PDAC, emerging evidence further suggests that lipid-derived mediators contribute to these interactions, providing a potential link between lipid metabolism, neural remodeling, and perineural invasion.

PDAC is characterized by extensive neural remodeling and frequent perineural invasion, involving reciprocal interactions among tumor cells, Schwann cells, neurons, CAFs, and immune cells within the perineural niche (207–211). Although direct evidence linking lipid metabolism to this neural niche remains limited, recent studies have identified specific lipid-derived mediators as regulators of tumor–neural crosstalk. Tumor-derived PGE_2_ promotes Schwann-cell dedifferentiation and increases the secretion of leukemia inhibitory factor (LIF) and a disintegrin and metalloproteinase with thrombospondin motif 1 (ADAMTS-1). LIF supports axonal growth and neural remodeling, whereas ADAMTS-1 promotes ECM degradation, collectively facilitating perineural invasion in PDAC (212). In parallel, CAF-derived sphingosine-1-phosphate (S1P) activates S1PR3/JNK/JUN signaling in PDAC cells and induces MAL-like protein (MALL) expression. MALL promotes syndecan-4 (SDC4) recycling to the plasma membrane, enhancing RhoA/p-MLC2-dependent amoeboid motility and tumor-cell responsiveness to Schwann cell-derived pleiotrophin, thereby strengthening directed neural invasion (213).

Emerging spatial profiling studies further suggest that lipid-associated neural regulation may extend beyond prostaglandin and sphingolipid signaling, with alterations in endocannabinoid metabolism identified within perineural invasion regions of human PDAC. However, the functional contribution of these metabolic changes to neural invasion and tumor progression remains to be established (214). Collectively, current evidence indicates that lipid-derived mediators can connect tumor and stromal metabolism with neural remodeling in PDAC. Nevertheless, mechanistic evidence remains largely centered on Schwann cell-associated perineural invasion, whereas the lipid metabolic regulation of sensory and autonomic neurons remains poorly understood.

Whether cancer-associated nerves or Schwann cells directly supply lipids to invading PDAC cells remains unclear. Cancer-associated Schwann cells in PDAC undergo c-Jun-dependent reprogramming toward a repair phenotype (215), while pancreatic cancer cells can induce ATG7-dependent autophagy in Schwann cells during perineural invasion (216). Evidence from peripheral nerve-injury models further shows that repair Schwann cells actively degrade myelin through myelinophagy, accompanied by LD accumulation and remodeling of myelin-derived CEs (217, 218). However, no direct lipid-tracing evidence currently demonstrates transfer of nerve- or Schwann-cell-derived lipids into invading PDAC cells. Thus, active myelin remodeling is biologically plausible in the PDAC neural niche, but whether it provides a metabolic lipid source for tumor cells remains to be established (**Figure 5**).

**Figure 5:**
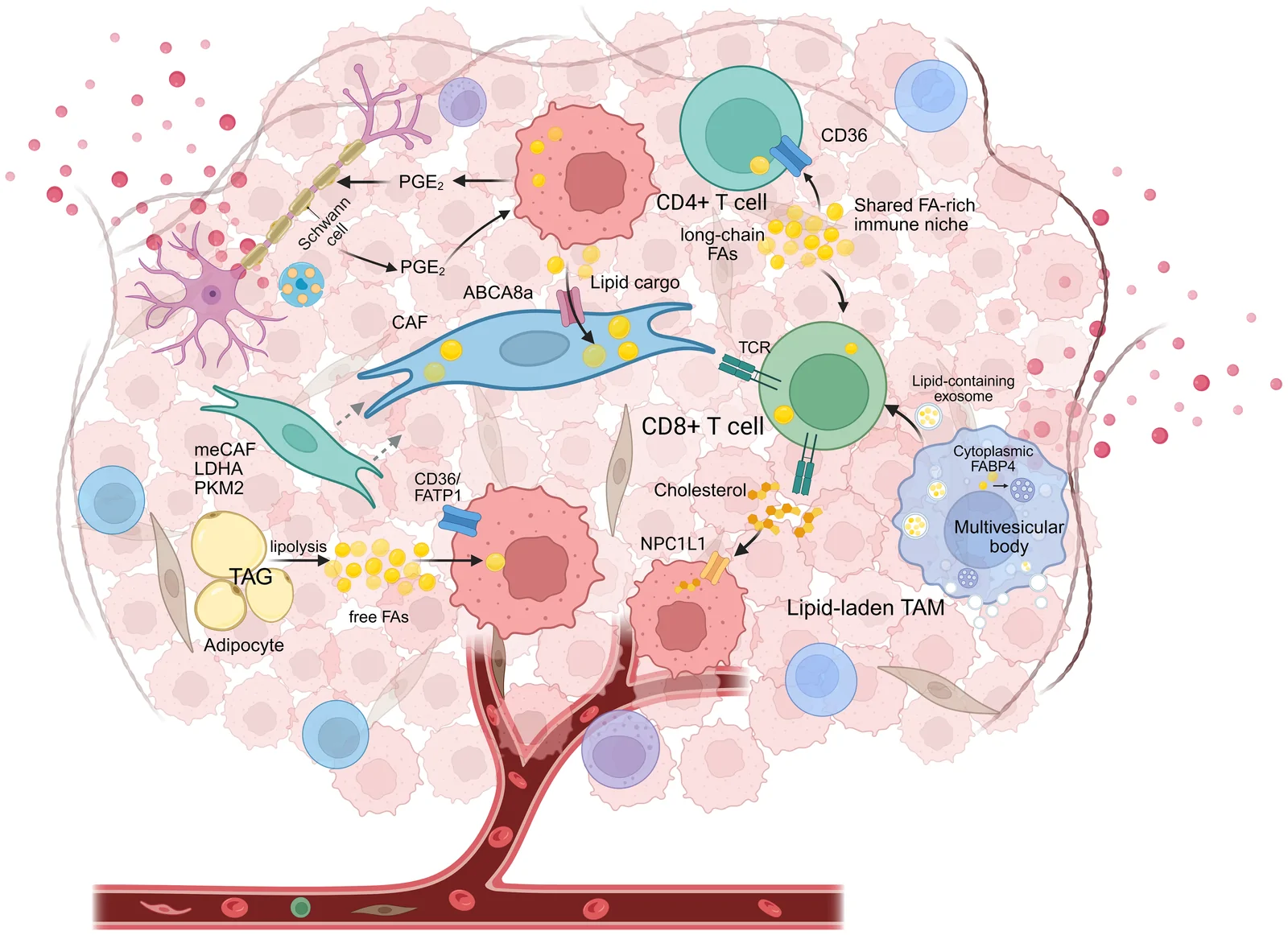
Multicellular lipid exchange network in the pancreatic ductal adenocarcinoma (PDAC) microenvironment. PDAC cells, cancer-associated fibroblasts (CAFs), metabolic CAFs (meCAFs), adipocytes, tumor-associated macrophages, T cells, and neural-associated cells form an interconnected lipid metabolic network. CAFs and adipocytes provide lipid substrates to tumor cells, whereas CD36- and fatty acid transport protein 1-associated pathways facilitate extracellular fatty acid (FA) acquisition. Within the immune compartment, shared FA-rich conditions, cholesterol competition, and lipid-containing extracellular vesicles link tumor cells and macrophages with CD4^+^ and CD8^+^ T-cell metabolism. Lipid-derived mediators such as prostaglandin E_2_ further connect PDAC cells with Schwann and neural cells. Together, these directional exchanges illustrate how lipid trafficking, metabolic competition, and intercellular signaling integrate malignant, stromal, immune, and neural compartments within the PDAC TME.

## 4 Circulating lipidomic signatures in PDAC

Beyond tumor-intrinsic lipid metabolic reprogramming, PDAC induces systemic lipid alterations that can be detected in blood. Circulating lipid profiles integrate multiple biological processes, including tumor-associated lipid uptake and utilization, adipose-tissue mobilization, hepatic lipoprotein metabolism, inflammation, nutritional status, and treatment exposure. Accordingly, they should not be regarded as a direct surrogate for the tumor lipidome but rather as a composite readout of tumor–host metabolic interactions.

### 4.1 Circulating lipidomic remodeling in PDAC and its clinical implications

Circulating lipid alterations may precede the clinical diagnosis of PDAC. A retrospective study showed declines in total cholesterol and LDL between 18 and 6 months before diagnosis, accompanied by reductions in body weight and subcutaneous fat (219). A prospective cohort study further revealed that before the occurrence of PDAC, there were already abnormalities in 43 lipid molecules and 5 FA indicators in the serum, covering multiple categories such as LPCs, PEs, PCs, glycerolipids, CEs, and SMs (220). These findings suggest that systemic lipid remodeling can emerge before clinical diagnosis and may reflect early tumor–host metabolic changes.

At clinical presentation, the most reproducible feature is not the alteration of a single lipid but a coordinated shift across several lipid classes. Clinical lipidomic studies have consistently reported changes in LPCs, PCs, PEs, SMs, Cers, and glycerolipids and multilipid classifiers have shown discriminatory potential for PDAC (221, 222). Recent targeted analyses further revealed molecular-species specificity: LPCs containing C16–C18 FAs and very-long-chain sphingolipids were generally reduced, whereas different PE subclasses showed divergent changes (223). A recent serum multi-omics study similarly identified increased diacylglycerols and reduced CEs, including altered CE(20:5), as components of the PDAC-associated circulating signature (224). Importantly, prospective studies suggest that such signatures may also be detectable in early-stage disease and in patients with low carbohydrate antigen 19-9 (CA19-9) secretion (225). The principal innovation of circulating lipidomics therefore lies in resolving molecular species and integrating complementary lipid signals, rather than relying on total cholesterol or any single lipid marker.

Circulating lipids may also provide prognostic and treatment-related information. Higher conventional plasma cholesterol and TAG levels have been associated with adverse clinicopathological features, reduced CD8^+^ T-cell infiltration, and poorer survival (182). In addition, lower exosomal LPC(16:0) and LPC(18:1) levels were observed in relapsing cases and were associated with shorter progression-free survival in a small longitudinal clinical study (226). Experimental lipidomics further demonstrated that PDAC-derived small EVs are enriched in sphingosine-1-phosphate, which promotes endothelial and fibroblast responses associated with tumor progression (227). Serum glycerolipids, phospholipids, and fatty acyls also differed between treatment-naïve and neoadjuvant FOLFIRINOX-treated patients, suggesting potential utility for treatment monitoring (228). Nevertheless, these prognostic and response-related findings require validation in larger, prospectively sampled cohorts.

### 4.2 Distinguishing PDAC from chronic pancreatitis through circulating lipidomic profiles

Differentiating PDAC from chronic pancreatitis (CP) is substantially more challenging than distinguishing PDAC from healthy individuals because both conditions are accompanied by pancreatic inflammation, fibrosis, exocrine dysfunction, and systemic metabolic alterations. Direct plasma comparisons have nevertheless identified modest but reproducible differences in selected SM and Cers between PDAC and CP (229). Because these differences are generally limited in magnitude, their diagnostic value is more likely to arise from combining several partially independent lipid signals than from a disease-specific alteration in a single lipid species.

This concept has been supported in increasingly clinically relevant cohorts. Serum SM 41:1 and Cer 41:1 were reduced in PDAC, including early-stage tumors, whereas their concentrations in CP were closer to those in healthy controls (222). A subsequent multicenter study involving 941 participants validated a single-platform, lipid-containing multimetabolite signature for differentiating PDAC from CP (230). More importantly, the prospective phase 4 METAPAC study evaluated these signatures in 1, 129 patients with clinically suspicious pancreatic lesions, including 113 patients with CP. The signatures showed higher specificity than CA19-9 alone, although sensitivity remained incomplete (231). These findings suggest that lipid-containing metabolite panels may be most useful as adjunctive tools alongside CA19-9 and pancreatic imaging rather than as replacements for pathological diagnosis.

The temporal interpretation of these differences requires caution. Similar lipid abnormalities have been observed in early and advanced stage PDAC, suggesting that some circulating lipid signals may emerge early without increasing progressively with tumor stage (222). Likewise, prospective serial sampling has not demonstrated a consistent lipid trajectory approaching PDAC diagnosis (220). Preliminary studies of exosomal LPCs and treatment-associated serum lipid changes suggest that selected lipid species may change with recurrence or treatment (226, 228), but no circulating lipid marker currently has a validated rate-of-change threshold for identifying malignant transformation in patients with CP. Future studies should therefore prioritize longitudinal lipidomic profiling in high-risk CP populations and integrate lipid signatures with CA19-9, imaging, and clinical risk factors.

## 5 Targeting lipid metabolism in PDAC

Lipid metabolic reprogramming contributes to PDAC growth, metastasis, and treatment resistance, providing new opportunities for metabolic intervention. Current strategies mainly target FA and cholesterol synthesis, lipid uptake and storage, FAO, lipid inflammatory mediators, and autophagy–lysosome-mediated recycling. Although several agents have shown antitumor potential in preclinical studies, their clinical benefits remain limited. This section therefore summarizes the therapeutic progress of major lipid metabolic targets and discusses their prospects for clinical translation and combination therapy (**Table 1**).

**Table 1:** Representative therapeutic strategies targeting lipid metabolism in pancreatic ductal adenocarcinoma.

Targeted process	Main target	Agent/compound	Pharmacological category	PDAC-related evidence	Study stage	Translational status	References
*De novo* fatty acid synthesis	Fatty acid synthase (FASN)	TVB-2640 (denifanstat)	FASN inhibitor	A phase I solid-tumor trial demonstrated target engagement and manageable toxicity; PDAC-specific efficacy remains unknown.	Phase I; advanced solid tumors	Clinical-stage	(233, 264)
		Omeprazole	Repurposed approved proton pump inhibitor	Preclinical FASN inhibition was reported, but no peer-reviewed PDAC efficacy data are available.	Early phase I	Approved/ repurposed	(234, 235)
		Orlistat	Repurposed approved anti-obesity drug	Inhibited PDAC-cell growth and enhanced gemcitabine activity in preclinical models.	Preclinical PDAC study	Approved/ repurposed	(29, 236)
		EGCG	Green tea-derived polyphenolic compound	Suppressed PDAC-cell growth and enhanced gemcitabine activity *in vitro*.	Phase I; as part of a combined dietary supplement	Preclinical tool	(238, 265)
	ATP citrate lyase (ACLY)	SB-204990	ACLY inhibitor	Reduced lipid abundance and viability in PDAC cells *in vitro*.	Preclinical PDAC study	Laboratory tool	(239)
	Acetyl-CoA carboxylase (ACC)	BAY ACC002	ACC inhibitor	Suppressed PDAC growth in cell, patient-derived and xenograft models.	Preclinical PDAC study	Preclinical tool	(22)
Fatty acid (FA) desaturation	Stearoyl-CoA desaturase (SCD)	A939572	SCD inhibitor	Inhibited PDAC cells, organoids and early murine pancreatic lesions.	Preclinical PDAC study	Preclinical tool	(69)
Cholesterol synthesis	3-Hydroxy-3-methyl-glutaryl- coenzyme A reductase (HMGCR)	Statins	Repurposed approved lipid-lowering drugs	A randomized phase II trial showed no clinical benefit; observational findings remain inconsistent.	Phase II	Approved/ repurposed	(95, 242, 244, 266–268)
Cholesterol uptake	NPC1L1	Ezetimibe	Repurposed approved cholesterol absorption inhibitor	Enhanced anti-PD-1 activity in PDAC cell and mouse models.	Preclinical PDAC study	Approved/ repurposed	(117)
Cholesterol depletion	HMGCR; NPC1L1; PCSK9	Atorvastatin+Ezetimibe+ Evolocumab	Multi-pathway cholesterol-lowering regimen	Explored with FOLFIRINOX in a small advanced-PDAC feasibility study.	Early phase I	Approved/ repurposed	(245)
Cholesterol esterification	Sterol O-acyltransferase (SOAT)	Avasimibe	SOAT inhibitor	Suppressed tumor growth and metastasis and enhanced gemcitabine activity in PDAC models.	Preclinical PDAC study	Discontinued	(129, 132)
Arachidonic acid-COX/PGE_2_ pathway	Cyclooxygenase-2 (COX-2)	Celecoxib	Selective COX-2 inhibitor	A phase II combination study showed no clear clinical benefit in advanced PDAC.	Phase II	Approved/ repurposed	(247, 269)
		Apricoxib	Selective COX-2 inhibitor	Preclinical sensitization did not translate into improved survival in a randomized phase II trial.	Phase II	Discontinued	(248, 249)
PGE_2_–EP4 signaling	Prostaglandin E receptor 4 (EP4)	E7046	EP4 antagonist	A first-in-human solid-tumor study demonstrated immunomodulatory activity; PDAC-specific efficacy remains unknown.	Phase I; advanced solid tumors	Clinical- stage	(250)
	EP4	L001	EP4 antagonist	Reduced PDAC migration, invasion and metastasis in cell and mouse models.	Preclinical PDAC study	Preclinical tool	(251)
5-Lipoxygenase (5-LOX/ leukotriene pathway)	5-LOX	Zileuton	5-LOX inhibitor	Reduced macrophage polarization, invasion and metastasis in preclinical PDAC models.	Preclinical PDAC study	Approved/ repurposed	(252)
4-LOX/Thromboxane A2 (TXA2)- 5-related pathway	5-LOX; TXA2 synthase	CV6504	Dual 5-LOX/TXA2 synthase inhibitor	A phase II PDAC study showed no objective responses.	Phase II	Discontinued	(253)
FA uptake	CD36	Sulfo-N-succinimidyl oleate (SSO)	CD36 inhibitor	Reduced lipid droplet accumulation and PDAC-cell migration *in vitro*.	Preclinical PDAC study	Laboratory tool	(147, 270)
Lipid transport	FABP4	HTS01037	FABP4 inhibitor	Suppressed PDAC growth and metastasis and enhanced gemcitabine activity in preclinical models.	Preclinical PDAC study	Preclinical tool	(254)
Lipid droplet storage	MTTP/TG synthesis	Lomitapide	Repurposed approved lipid-lowering drug	Enhanced FOLFIRINOX efficacy in patient-derived xenograft and genetically engineered PDAC models.	Preclinical PDAC study	Approved/ repurposed	(20)
Fatty acid oxidation (FAO)	CPT1A/ FAO	Etomoxir	CPT1A inhibitor	Reduced oxidative metabolism, stem-like properties and chemotherapy tolerance in PDAC models.	Preclinical PDAC study	Laboratory tool	(11, 271)
	3-ketoacyl-CoA thiolase	Ranolazine	Repurposed approved antianginal drug	Reduced stem-like properties and enhanced gemcitabine activity in PDAC models.	Preclinical PDAC study	Approved/repurposed	(11)
Lipid peroxidation/ ferroptosis	Iron-dependent lipid peroxidation	Artesunate	Repurposed clinically used antimalarial drug	Induced ferroptosis and suppressed KRAS-mutant PDAC growth in preclinical models.	Preclinical PDAC study	Approved/repurposed	(255)
	STING1/ TMEM173–ALOX5-related axis	Zalcitabine	Repurposed clinically used antiviral drug	Induced mitochondrial DNA stress and ferroptosis in PDAC cells and xenografts.	Preclinical PDAC study	Discontinued	(256)
Autophagy–lysosome-mediated lipid recycling	Lysosome	Hydroxy-chloroquine	Approved antimalarial drug	Randomized PDAC studies showed response-related effects but no established overall-survival benefit.	Phase II	Approved/repurposed	(257, 258, 272)
Lysosomal lipid homeostasis	PIKfyve	Apilimod	PIKfyve inhibitor	Sensitized PDAC cells, organoids and mouse models to KRAS or MEK inhibition.	Preclinical PDAC study	Clinical-stage	(259)
		ESK981	PIKfyve inhibitor	Enhanced KRAS- or MEK-targeted therapy in murine and patient-derived PDAC models.	Preclinical PDAC study	Clinical-stage	(259, 273)
Transcriptional regulation of lipid metabolism	SREBP1	Fatostatin	SREBP inhibitor	Reduced lipogenic signaling and PDAC growth in cell and xenograft models.	Preclinical PDAC study	Laboratory tool	(260)
	SREBF1/ FASN/SCD-related axis	Yarrow CO_2_ supercritical extract	Natural product-derived extract	Reduced lipid accumulation, invasion and xenograft growth in preclinical PDAC models.	Preclinical PDAC study	Laboratory preparation	(261)
	LXR	GAC0001E5	LXR inverse agonist/ degrader	Reduced LXR activity and PDAC-cell proliferation, primarily *in vitro*.	Preclinical PDAC study	Preclinical tool	(262)

### 5.1 From lipid metabolic abnormalities to therapeutic intervention

Despite substantial advances in surgery, systemic therapy, radiotherapy, targeted therapy, and immunotherapy, the prognosis of PDAC remains poor (232). Lipid metabolic reprogramming is increasingly recognized not as a passive consequence of rapid proliferation but as an active determinant of tumor growth, metastatic dissemination, immune evasion, and treatment resistance (15). Accordingly, interventions that disrupt lipid synthesis, uptake, trafficking, storage, oxidation, or lysosomal recycling have attracted increasing interest (9). Many of these strategies do not directly kill tumor cells as conventional cytotoxic drugs do. Instead, they aim to reduce the metabolic flexibility that helps PDAC cells survive under nutrient shortage, hypoxia, and treatment stress. This section evaluates these agents based on their molecular targets, preclinical evidence, clinical development, and major limits to clinical use.

### 5.2 Agents targeting *de novo* fatty acid metabolism

FA synthesis and desaturation were among the first lipid metabolic processes considered for therapeutic targeting in PDAC, with FASN, ACLY, ACC, and SCD emerging as major enzymatic candidates. The oral FASN inhibitor TVB-2640 entered a phase I trial in patients with advanced solid tumors, where it was evaluated both as monotherapy and in combination with taxanes. The trial confirmed pharmacologic target engagement and reported an overall manageable safety profile, but it did not provide sufficient evidence to define efficacy specifically in PDAC (233). Proton pump inhibitors have also attracted interest because preclinical studies suggest that they can interfere with the thioesterase domain of FASN (234). High-dose omeprazole was subsequently examined in a presurgical early-phase biomarker study in exocrine PDAC; the study was terminated after enrolling 25 participants, and no outcome data have been posted (235). Orlistat, a gastrointestinal lipase inhibitor that can also covalently inhibit the FASN thioesterase domain, reduced proliferation and promoted apoptosis in PANC-1 cells (236). Although suppression of *de novo* lipid synthesis has been reported to enhance gemcitabine activity in preclinical PDAC models (29), these observations do not yet support the clinical use of orlistat as an anticancer agent. Epigallocatechin gallate (EGCG) is another nonselective natural compound with reported FASN-inhibitory activity (237). In PDAC, EGCG was included as one component of a multi-ingredient dietary supplement administered with gemcitabine, nab-paclitaxel, and metformin in a pilot phase I study; consequently, any clinical effect cannot be attributed specifically to EGCG or to FASN inhibition (238).

Other enzymes remain at the preclinical stage. Pharmacological or genetic inhibition of ACLY reduced viability and lipid abundance in PANC-1 and CFPAC-1 cells (239). The ACC inhibitor BAY ACC002 attenuated WNT and Hedgehog signaling and suppressed growth in pancreatic cancer cell lines and patient-derived models (22). The SCD1 inhibitor A939572 activated the unfolded protein response. It impaired the growth of PDAC organoids, early murine pancreatic lesions, and PANC-1 cells, although overt cell death was not observed in PANC-1 cells (69). These results suggest that FA synthesis and desaturation may be useful therapeutic targets, but the response differs between models, drug selectivity remains limited, and PDAC-specific clinical evidence is still lacking.

### 5.3 Agents targeting cholesterol metabolism

Cholesterol metabolism can be therapeutically targeted at several levels, including the mevalonate pathway, extracellular cholesterol uptake, and cholesterol esterification (240). Within the mevalonate pathway, statins inhibit HMGCR, the rate-limiting enzyme of mevalonate synthesis, thereby reducing the production of both cholesterol and non-sterol isoprenoid intermediates. However, clinical results have been less encouraging. In a randomized, double-blind phase II trial, adding simvastatin to gemcitabine did not improve clinical outcomes in patients with advanced PDAC (241). By contrast, retrospective analyses and a meta-analysis have suggested a possible survival advantage among statin users, particularly in patients who underwent tumor resection. These observational findings should be interpreted cautiously because residual confounding cannot be excluded, and a consistent benefit has not been demonstrated in advanced pancreatic cancer (242). Other epidemiological meta-analyses mainly examined the risk of developing PDAC rather than treatment response, so they should not be used as evidence of therapeutic benefit (243). A phase II study is evaluating valproic acid and simvastatin in combination with gemcitabine/nab-paclitaxel-based treatment in metastatic PDAC, but a trial protocol does not itself establish clinical benefit (244). The NPC1L1 inhibitor ezetimibe has recently shown preclinical immunometabolic activity. NPC1L1-expressing PDAC cells depleted cholesterol from CD8^+^ T cells and suppressed T-cell receptor signaling, whereas ezetimibe preserved T-cell cholesterol levels and enhanced the response to PD-1 blockade in mouse models (117). These findings are still preclinical. An early-phase feasibility study tested atorvastatin, ezetimibe, and the PCSK9 inhibitor evolocumab with FOLFIRINOX; only three participants were enrolled, and no results have been reported, precluding conclusions regarding efficacy (245). Avasimibe is a pharmacological inhibitor of SOAT activity. Genetic or pharmacological inhibition of cholesterol esterification reduced CE accumulation, proliferation, orthotopic tumor growth, and metastasis in PDAC models (129). Avasimibe also enhanced gemcitabine activity in gemcitabine-resistant PDAC models (132). Overall, cholesterol metabolism is experimentally tractable, but its clinical translation remains constrained by alternative cholesterol acquisition, tumor heterogeneity, and limited prospective clinical evidence, highlighting the need for biomarker-guided patient selection and rational combination strategies (114, 128).

### 5.4 Agents targeting arachidonic acid metabolism

AA released from membrane phospholipids is metabolized through cyclooxygenase (COX), lipoxygenase (LOX), and related pathways into prostaglandins, leukotrienes, and other bioactive mediators that can shape inflammation, immunosuppression, invasion, metastasis, and treatment response in PDAC (246). Clinical translation, however, has been disappointing. In a phase II study, Celecoxib combined with gemcitabine did not yield a clear improvement in clinical outcomes (247). Apricoxib enhanced gemcitabine/erlotinib activity in molecularly selected preclinical PDAC models (248); however, in the randomized phase II APRiCOT-P study, median progression-free and overall survival were similar between the apricoxib and placebo groups, providing no convincing signal of efficacy (249). Downstream blockade of PGE_2_ signaling has also been explored. The prostaglandin E receptor 4 (EP4) antagonist E7046 demonstrated manageable safety and immunomodulatory pharmacodynamic activity in a first-in-human study in advanced solid tumors, but PDAC-specific efficacy was not established (250). In preclinical PDAC models, the EP4 antagonist L001 suppressed PGE_2_-induced migration, invasion, and metastasis (251). The approved 5-LOX inhibitor zileuton reduced invasion and metastasis by modulating TAM polarization in preclinical models (252). By contrast, the lipoxygenase and thromboxane A_2_ synthase inhibitor CV6504 produced no objective responses in a phase II study, although stable disease was observed in a subset of patients (253). Thus, despite clear biological relevance, targeting AA-derived inflammatory mediators has not yet yielded an established therapeutic strategy for PDAC.

### 5.5 Agents targeting lipid uptake and utilization

PDAC cells can compensate for restricted lipid synthesis by scavenging extracellular FAs, transporting them through intracellular binding proteins, storing them as TAGs in LDs, and oxidizing them in mitochondria. These processes provide several candidate targets, although the supporting evidence is still predominantly preclinical. Inhibition of CD36 with sulfo-N-succinimidyl oleate reduced adipocyte-derived FA-driven LD formation and PDAC cell migration (147). But this compound is mainly used as a research tool and is not a clinically validated selective CD36 inhibitor. The FABP4 inhibitor HTS01037 reduced PDAC growth, invasion, and metastasis in cell and mouse models. It also increased the antitumor effect of gemcitabine (254). Under cystine-limiting conditions, lomitapide disrupted adaptive TAG synthesis and LD formation, and enhanced the activity of FOLFIRINOX in patient-derived and genetically engineered PDAC models (20). FAO may represent a downstream vulnerability, especially in pancreatic cancer stem-like cells. Pharmacological FAO inhibition reduced oxidative phosphorylation, tumor-initiating ability, and chemotherapy tolerance, while ranolazine enhanced gemcitabine activity *in vitro* and *in vivo* (11). Another distinct strategy is to increase lipid peroxidation instead of blocking lipid use. Artesunate induced ferroptotic cell death in KRAS-mutant PDAC models through iron-dependent lipid peroxidation, while GRP78 reduced this effect (255). Zalcitabine induced mitochondrial DNA stress and STING1-dependent, autophagy-associated ferroptosis in PDAC cells and xenografts (256). These repurposing observations identify potential vulnerabilities but remain insufficient for clinical adoption without prospective validation and mechanism-based patient selection.

### 5.6 Autophagy and lipid recycling

The autophagy–lysosome pathway provides PDAC cells with recycled intracellular substrates and damaged organelle components, helping them adapt to nutrient limitation and therapeutic stress. Hydroxychloroquine interferes with lysosomal acidification and blocks late-stage autophagic degradation, although its action is not specific to lipid metabolism. In a randomized phase II study, adding hydroxychloroquine to gemcitabine and nab-paclitaxel increased the objective response rate but failed to extend overall survival in advanced PDAC (257). In the neoadjuvant setting, high-dose hydroxychloroquine combined with gemcitabine and nab-paclitaxel was associated with improvements in several pathological response endpoints, whereas clear benefits in overall or recurrence-free survival were not observed (258). Phosphoinositide kinase, FYVE-type zinc finger containing (PIKfyve) has recently been studied as a regulator of lysosomal function and lipid homeostasis. Inhibition of PIKfyve with apilimod or ESK981 reduced lysosomal and autophagic flux, increased intracellular lipid retention, and activated an SREBP-dependent lipogenic program. This increased the dependence of PDAC cells on *de novo* lipid synthesis. Combining PIKfyve inhibition with the KRAS-G12D inhibitor MRTX1133 or the MEK inhibitor trametinib produced strong antitumor effects in several preclinical PDAC models, including tumor regression or complete tumor loss in some models (259). But previous clinical use of apilimod and ESK981 in other diseases does not prove that these combinations are effective in PDAC. Other studies showed that restriction of extracellular lipids or suppression of ACSL3 activated protective autophagy. When autophagy was also inhibited, tumor growth was further reduced, and apoptosis increased in PDAC models (141). Collectively, autophagy–lysosome-directed approaches appear more plausible as rational combination partners than as stand-alone lipid-targeted treatments.

### 5.7 Lipid transcriptional regulation

Transcriptional regulators can affect multiple lipid pathways simultaneously, including FA synthesis, cholesterol metabolism, redox homeostasis, and membrane homeostasis. But this broad effect may also raise concerns regarding selectivity and systemic toxicity. SREBPs are central regulators of lipogenic gene expression. The SREBP inhibitor fatostatin suppressed PDAC cell proliferation and xenograft growth and reduced *GPX4* transcription, thereby promoting ferroptotic features in preclinical models (260). Because fatostatin can indirectly affect SREBP processing, further target-validation studies are needed. Yarrow supercritical CO_2_ extract reduced *SREBF1* expression and its downstream targets *FASN* and *SCD*, decreased neutral lipid abundance, and suppressed PDAC cell invasion and xenograft growth (261). Nevertheless, as a complex botanical preparation, it cannot be considered a selective SREBP inhibitor. Liver X receptors (LXRs) are nuclear receptors that regulate cholesterol transport, lipid metabolism, and inflammatory responses. The LXR inverse agonist and degrader GAC0001E5 inhibited LXR transcriptional activity, reduced LXR protein abundance, and suppressed PDAC cell proliferation; related mechanistic studies also linked its activity to impaired glutamine metabolism and oxidative stress (262). By comparison, the combination of betulinic acid and mithramycin A suppressed PDAC growth through broad inhibition of Sp1-dependent proliferation, invasion, and angiogenesis rather than through a demonstrated lipid-specific dependency (263). For this reason, this combination is better viewed as an upstream transcriptional strategy than as a direct lipid metabolism-targeted treatment. Current evidence for transcriptional regulators is still mainly preclinical, and drug specificity remains a significant challenge.

## 6 Discussion

PDAC remains one of the most challenging tumors to treat, largely because it is often diagnosed at an advanced stage and is characterized by rapid progression, early metastasis, and therapeutic resistance (4, 7). These clinical challenges are closely linked to extensive remodeling of lipid metabolism, which allows PDAC cells to survive in hypoxic, nutrient-poor and poorly perfused environments (8, 9, 266). FAs and cholesterol provide substrates for membrane biogenesis, energy production, protein lipidation, and oncogenic signaling, while also influencing redox homeostasis, ferroptosis sensitivity, invasion, metastasis, and treatment response (9, 266). In this review, we summarize tumor cell intrinsic FA and cholesterol metabolism, discuss their bidirectional interactions with the TME, and assess emerging diagnostic and therapeutic strategies directed at these metabolic nodes.

One major challenge in targeting lipid metabolism in PDAC is the ability of tumor cells to adaptively maintain FA supply and composition. PDAC cells can draw on several partly interchangeable routes, including *de novo* synthesis through FASN and ACC (22, 27, 84), uptake of extracellular FAs through CD36 and FATPs (30, 31), with macropinocytosis providing an additional route for lipid acquisition (33). SCD1-dependent desaturation further adjusts FA composition and supports lipid homeostasis (34). LDs connect these processes by storing excess lipids and releasing them through neutral lipolysis or lipophagy when metabolic demand changes (16, 133, 140). Consequently, inhibiting a single pathway may redirect FA flux rather than produce a sustained reduction in intracellular lipid availability (21, 25, 141, 266). For example, suppression of endogenous FA synthesis may increase reliance on extracellular lipid uptake (141), whereas nutrient or lipid restriction may shift tumor cells toward autophagy-mediated lipid recycling and FAO (18). Accordingly, compared with inhibition of a single metabolic enzyme, concurrent blockade of lipid synthesis and compensatory pathways that sustain FA availability may offer greater therapeutic efficacy.

Cholesterol metabolism shows similar plasticity. PDAC cells can acquire cholesterol either through *de novo* synthesis mediated by HMGCR and SQLE or through extracellular uptake via NPC1L1 and LDLR (24, 108, 114, 117). Excess cholesterol can be esterified by SOAT1 and stored as CEs (128, 129). Together, these processes help maintain membrane organization and lipid-raft signaling (24, 129, 274). Thus, blocking cholesterol synthesis alone may be offset by increased cholesterol uptake or storage, which may partly explain why statins have not consistently improved survival in unselected patients with PDAC (242, 268). Therefore, identifying tumors with a clear dependence on cholesterol metabolism may be important for patients most likely to benefit from such strategies.

The TME further shapes lipid metabolic adaptation in PDAC. When tumor cell lipid synthesis is insufficient, CAFs can supply FAs and lysophospholipids that support membrane production, energy metabolism, and treatment tolerance (21, 146, 152). CAF-derived EVs can also limit ACSL4-dependent lipid peroxidation, thereby reducing ferroptosis and promoting gemcitabine resistance (165). In immune cells, however, long-chain FAs and excess cholesterol can impair mitochondrial function in CD8^+^ T cells, favor ferroptosis or exhaustion, and weaken cytotoxic activity (145, 174, 175). By contrast, CD36-mediated FA uptake can support Treg metabolism, whereas FABP4 and FABP7 can promote lipid accumulation and an immunosuppressive phenotype in macrophages (183, 188, 191). These macrophages can further transfer lipids to tumor cells, suppress CD8^+^ T cell activity, and contribute to formation of a premetastatic niche in the liver (191). These findings indicate that lipid metabolism in PDAC is not confined to tumor cells but is coordinated across TME. Accordingly, future therapeutic strategies should not only target tumor-intrinsic lipid metabolism but also aim to restore lipid homeostasis within the TME.

Beyond therapeutic targeting, circulating lipidomics offers a potential route for translating lipid metabolic alterations into diagnostic tools. Lipid-containing multimetabolite panels have shown promise in distinguishing PDAC from CP, supporting their evaluation alongside CA19-9 and pancreatic imaging (229–231). However, circulating lipid profiles reflect both tumor-associated changes and systemic influences, including nutritional status, inflammation, and treatment exposure. Diagnostic discrimination therefore does not establish tumor-specific metabolic dependence or suitability for selecting lipid-targeted therapy. Future studies should prioritize prospective validation in clinically relevant populations, assay standardization, and longitudinal sampling to determine whether these signatures improve clinical decisions beyond existing tests.

Despite these challenges, several emerging approaches suggest that lipid metabolism remains a therapeutically relevant vulnerability in PDAC. Combining PIKfyve inhibition with KRAS-G12D or MEK inhibition has shown substantial preclinical antitumor activity (259). In a spontaneous KPC model of PDAC, ESK981 plus the MEK inhibitor selumetinib extended median survival from 11 to 58.5 days and induced tumor regression in five of six mice (259). Targeting LD storage or FAO with lomitapide, ranolazine, or related agents may likewise enhance the activity of FOLFIRINOX or gemcitabine in metabolically adaptive tumors. By suppressing TAG synthesis and LD formation, lomitapide enhanced the antitumor effect of FOLFIRINOX in two PDAC patient-derived xenograft models. Tumor growth inhibition increased from 52% and 16% to 78% and 77%, while median survival in a KPC model was prolonged from 65 to 121 days (20). Inhibition of FASN, ACC, or SCD may disrupt membrane synthesis and redox adaptation; combining these approaches with chemotherapy or ferroptosis induction may further limit compensatory lipid use (22, 29, 34). Increased SREBP1 activity may also reshape lipid metabolism and alter treatment response (260, 275). Finally, targeting CD36, FABP4/FABP7, EP4, or 5-LOX-dependent lipid signaling may help counter lipid-associated immunosuppression mediated by Treg cells and TAMs (183, 188, 191, 252, 276). Together, these findings support further development of lipid-directed combination strategies to improve treatment efficacy in PDAC.

Overall, lipid metabolism provides opportunities for both biomarker development and therapeutic intervention in PDAC. Clinical translation will require distinguishing metabolic associations from functional dependencies, identifying treatment-induced compensation, and validating diagnostic panels and combination therapies in appropriately selected patient populations.

## References

[1] Klein AP . Pancreatic cancer epidemiology: Understanding the role of lifestyle and inherited risk factors. Nat Rev Gastroenterol Hepatol. (2021) 18:493–502. doi: 10.1038/s41575-021-00457-x 34002083 PMC9265847

[2] Leiphrakpam PD Chowdhury S Zhang M Bajaj V Dhir M Are C . Trends in the global incidence of pancreatic cancer and a brief review of its histologic and molecular subtypes. J Gastrointestinal Cancer. (2025) 56:71. doi: 10.1007/s12029-025-01183-2 39992560

[3] Sung H Filho AM Laversanne M Ferlay J Siegel RL Soerjomataram I . Global cancer statistics 2024: Globocan estimates of incidence and mortality worldwide for 34 cancers in 186 countries. Ca. (2026) 76:e70090. doi: 10.3322/caac.70090 42417444 PMC13343830

[4] Wood LD Canto MI Jaffee EM Simeone DM . Pancreatic cancer: Pathogenesis, screening, diagnosis and treatment. Gastroenterology. (2022) 163:386–402.e1. doi: 10.1053/j.gastro.2022.03.056 35398344 PMC9516440

[5] Moutinho-Ribeiro P Iglesias-Garcia J Gaspar R Macedo G . Early pancreatic cancer - the role of endoscopic ultrasound with or without tissue acquisition in diagnosis and staging. Digestive Liver Dis Off J Ital Soc Gastroenterol Ital Assoc For Study Liver. (2019) 51:4–9. doi: 10.1016/j.dld.2018.09.027 30337098

[6] Yang J Xu R Wang C Qiu J Ren B You L . Early screening and diagnosis strategies of pancreatic cancer: A comprehensive review. Cancer Commun. (2021) 41:1257–74. doi: 10.1002/cac2.12204 34331845 PMC8696234

[7] Hu ZI O’Reilly EM . Therapeutic developments in pancreatic cancer. Nat Rev Gastroenterol Hepatol. (2023) 21:7–24. doi: 10.1038/s41575-023-00840-w 37798442

[8] Encarnación-Rosado J Kimmelman AC . Harnessing metabolic dependencies in pancreatic cancers. Nat Rev Gastroenterol Hepatol. (2021) 18:482–92. doi: 10.1038/s41575-021-00431-7 PMC8249349 33742165

[9] Yin X Xu R Song J Ruze R Chen Y Wang C . Lipid metabolism in pancreatic cancer: Emerging roles and potential targets. Cancer Commun. (2022) 42:1234–56. doi: 10.1002/cac2.12360 36107801 PMC9759769

[10] Wen Y Xing X Harris JW Zaytseva YY Mitov MI Napier DL . Adipocytes activate mitochondrial fatty acid oxidation and autophagy to promote tumor growth in colon cancer. Cell Death Dis. (2017) 8:e2593. doi: 10.1038/cddis.2017.21 28151470 PMC5386470

[11] Mascaraque M Courtois S Royo-García A Barneda D Stoian AM Villaoslada I . Fatty acid oxidation is critical for the tumorigenic potential and chemoresistance of pancreatic cancer stem cells. J Transl Med. (2024) 22:797. doi: 10.1186/s12967-024-05598-6 39198858 PMC11351511

[12] Vogel FCE Chaves-Filho A Schulze A . Lipids as mediators of cancer progression and metastasis. Nat Cancer. (2024) 5:16–29. doi: 10.1038/s43018-023-00702-z 38273023

[13] Corn KC Windham M Rafat M . Lipids in the tumor microenvironment: From cancer progression to treatment. Prog Lipid Res. (2020) 80:101055. doi: 10.1016/j.plipres.2020.101055 32791170 PMC7674189

[14] Ishida CT Myers SL Kubota CS Shao W McGuire MR Liu C . Srebp-dependent regulation of lipid homeostasis is required for progression and growth of pancreatic ductal adenocarcinoma. Cancer Res Commun. (2024) 4:2539–52. doi: 10.1158/2767-9764.crc-24-0120 39240063 PMC11444119

[15] Zhang Y Yang Z Liu Y Pei J Li R Yang Y . Targeting lipid metabolism: Novel insights and therapeutic advances in pancreatic cancer treatment. Lipids Health Dis. (2025) 24:12. doi: 10.1186/s12944-024-02426-0 39806478 PMC11727729

[16] Rozeveld CN Johnson KM Zhang L Razidlo GL . Kras controls pancreatic cancer cell lipid metabolism and invasive potential through the lipase Hsl. Cancer Res. (2020) 80:4932–45. doi: 10.1158/0008-5472.Can-20-1255 32816911 PMC7669720

[17] Lin Y Zhang X Wang Y Yao W . Lpcat2-mediated lipid droplet production supports pancreatic cancer chemoresistance and cell motility. Int Immunopharmacol. (2024) 139:112681. doi: 10.1016/j.intimp.2024.112681 39068758

[18] Thakur R Wang D Hu T He C Zhao J Suresh V . Kras signaling inhibition induces a targetable metabolic dependency on lipophagy-dependent fatty acid oxidation in pancreatic cancer. Cancer Res. (2026) 86:3519–36. doi: 10.1158/0008-5472.can-24-1984 42054558 PMC13246267

[19] Gao J Zhang Z Liu Y Zhang Z Wang M Gong A . Stearoyl-Coa desaturase 1 potentiates hypoxic plus nutrient-deprived pancreatic cancer cell ferroptosis resistance. Oxid Med Cell Longevity. (2021) 2021:6629804. doi: 10.1155/2021/6629804 33868572 PMC8032529

[20] Li Y Li Z Li Q Sun D Ni B Tan M . Adaptation to cystine limitation stress confers a targetable lipid metabolism vulnerability in pancreatic ductal adenocarcinoma. Nat Commun. (2025) 17:1343. doi: 10.1038/s41467-025-68099-0 41476081 PMC12872613

[21] Han X Burrows M Kim LC Xu J Vostrejs WP Ngoc TH . Cancer-associated fibroblasts maintain critical pancreatic cancer cell lipid homeostasis in the tumor microenvironment. Cell Rep. (2024) 43:114972. doi: 10.1016/j.celrep.2024.114972 39535921 PMC11648993

[22] Petrova E Scholz A Paul J Sturz A Haike K Siegel F . Acetyl-Coa carboxylase inhibitors attenuate Wnt and Hedgehog signaling and suppress pancreatic tumor growth. Oncotarget. (2016) 8:48660–70. doi: 10.18632/oncotarget.12650 27750213 PMC5564715

[23] Kubota CS Myers SL Seppälä TT Burkhart RA Espenshade PJ . *In vivo* Crispr screening identifies geranylgeranyl diphosphate as a pancreatic cancer tumor growth dependency. Mol Metab. (2024) 85:101964. doi: 10.1016/j.molmet.2024.101964 38823776 PMC11217740

[24] Xu R Song J Ruze R Chen Y Yin X Wang C . Sqle promotes pancreatic cancer growth by attenuating Er stress and activating lipid rafts-regulated Src/Pi3k/Akt signaling pathway. Cell Death Dis. (2023) 14:497. doi: 10.1038/s41419-023-05987-7 37542052 PMC10403582

[25] Koundouros N Poulogiannis G . Reprogramming of fatty acid metabolism in cancer. Br J Cancer. (2019) 122:4–22. doi: 10.1038/s41416-019-0650-z 31819192 PMC6964678

[26] Yoon H Shaw JL Haigis MC Greka A . Lipid metabolism in sickness and in health: Emerging regulators of lipotoxicity. Mol Cell. (2021) 81:3708–30. doi: 10.1016/j.molcel.2021.08.027 34547235 PMC8620413

[27] Bian Y Yu Y Wang S Li L . Up-regulation of fatty acid synthase induced by Egfr/Erk activation promotes tumor growth in pancreatic cancer. Biochem Biophys Res Commun. (2015) 463:612–7. doi: 10.1016/j.bbrc.2015.05.108 26043686

[28] Yang Y Liu H Li Z Zhao Z Yip-Schneider M Fan Q . Role of fatty acid synthase in gemcitabine and radiation resistance of pancreatic cancers. Int J Biochem Mol Biol. (2011) 2:89–98. PMC3039422 21331354

[29] Tadros S Shukla SK King RJ Gunda V Vernucci E Abrego JM . De novo lipid synthesis facilitates gemcitabine resistance through endoplasmic reticulum stress in pancreatic cancer. Cancer Res. (2017) 77:5503–17. doi: 10.1158/0008-5472.can-16-3062 28811332 PMC5645242

[30] Kubo M Gotoh K Eguchi H Kobayashi S Iwagami Y Tomimaru Y . Impact of Cd36 on chemoresistance in pancreatic ductal adenocarcinoma. Ann Surg Oncol. (2019) 27:610–9. doi: 10.1245/s10434-019-07927-2 31605325

[31] Xu Z Du J Jiang W Martin TA . Slc27, solute carrier 27 family, a long-chain fatty acid membrane transporters, in human cancers. Front Cell Dev Biol. (2026) 14. doi: 10.3389/fcell.2026.1839021 42440857 PMC13333737

[32] Tang R Xu J Wang W Meng Q Shao C Zhang Y . Targeting neoadjuvant chemotherapy-induced metabolic reprogramming in pancreatic cancer promotes anti-tumor immunity and chemo-response. Cell Rep Med. (2023) 4:101234. doi: 10.1016/j.xcrm.2023.101234 37852179 PMC10591062

[33] Chen F Tang H Lin J Xiang L Lu Y Kang R . Macropinocytosis inhibits alkaliptosis in pancreatic cancer cells through fatty acid uptake. Carcinogenesis. (2024) 45:953–64. doi: 10.1093/carcin/bgae045 39008332

[34] Ye Z Zhuo Q Hu Q Xu X Liu M Zhang Z . Fbw7-Nra41-Scd1 axis synchronously regulates apoptosis and ferroptosis in pancreatic cancer cells. Redox Biol. (2021) 38:101807. doi: 10.1016/j.redox.2020.101807 PMC7710650 33271455

[35] Xiao Q Lan Z Zhang S Ren H Wang S Wang P . Overexpression of Znf488 supports pancreatic cancer cell proliferation and tumorigenesis through inhibition of ferroptosis via regulating Scd1-mediated unsaturated fatty acid metabolism. Biol Direct. (2023) 18:77. doi: 10.1186/s13062-023-00421-6 37986084 PMC10658979

[36] Kamphorst JJ Cross JR Fan J de Stanchina E Mathew R White EP . Hypoxic and Ras-transformed cells support growth by scavenging unsaturated fatty acids from lysophospholipids. Proc Natl Acad Sci USA. (2013) 110:8882–7. doi: 10.1073/pnas.1307237110 23671091 PMC3670379

[37] Buckley D Duke GM Heuer TS O’Farrell M Wagman AS McCulloch W . Fatty acid synthase - modern tumor cell biology insights into a classical oncology target. Pharmacol Ther. (2017) 177:23–31. doi: 10.1016/j.pharmthera.2017.02.021 28202364

[38] Chirala SS Wakil SJ . Structure and function of animal fatty acid synthase. Lipids. (2004) 39:1045–53. doi: 10.1007/s11745-004-1329-9 15726818

[39] Walter K Hong SM Nyhan SM Canto MI Fedarko NS Klein AP . Serum fatty acid synthase as a marker of pancreatic neoplasia. Cancer Epidemiology Biomarkers Prev A Publ Am Assoc For Cancer Research Cosponsored by Am Soc Prev Oncol. (2009) 18:2380–5. doi: 10.1158/1055-9965.epi-09-0144 19723916 PMC2860720

[40] Maekawa H Ito T Orita H Sato K Wada R . Clinicopathological significance of fatty acid synthase expression in pancreatic ductal carcinoma. Int J Hepatobiliary Pancreat Dis. (2013) 3:10–6. doi: 10.5348/ijhpd-2013-11-OA-2

[41] Tian S Li P Sheng S Jin X . Upregulation of pyruvate kinase M2 expression by fatty acid synthase contributes to gemcitabine resistance in pancreatic cancer. Oncol Lett. (2018) 15:2211–7. doi: 10.3892/ol.2017.7598 29434927 PMC5777098

[42] Hunkeler M Hagmann A Stuttfeld E Chami M Guri Y Stahlberg H . Structural basis for regulation of human acetyl-Coa carboxylase. Nature. (2018) 558:470–4. doi: 10.1038/s41586-018-0201-4 29899443

[43] Wakil SJ Abu-Elheiga LA . Fatty acid metabolism: Target for metabolic syndrome. J Lipid Res. (2009) 50 Suppl:S138–43. doi: 10.1194/jlr.R800079-JLR200 19047759 PMC2674721

[44] Abu-Elheiga L Brinkley WR Zhong L Chirala SS Woldegiorgis G Wakil SJ . The subcellular localization of acetyl-Coa carboxylase 2. PNAS. (2000) 97:1444–9. doi: 10.1073/pnas.97.4.1444 10677481 PMC26453

[45] Schreurs M Kuipers F Leij F . Regulatory enzymes of mitochondrial β‐oxidation as targets for treatment of the metabolic syndrome. Obes Rev. (2010) 11:380–8. doi: 10.1111/j.1467-789x.2009.00642.x 19694967

[46] Röhrig F Schulze A . The multifaceted roles of fatty acid synthesis in cancer. Nat Rev Cancer. (2016) 16:732–49. doi: 10.1038/nrc.2016.89 27658529

[47] Foster DW . Malonyl-Coa: The regulator of fatty acid synthesis and oxidation. J Clin Invest. (2012) 122:1958–9. doi: 10.1172/jci63967 22833869 PMC3366419

[48] Ngo J Choi DW Stanley IA Stiles L Molina AJA Chen P-H . Mitochondrial morphology controls fatty acid utilization by changing Cpt1 sensitivity to malonyl‐Coa. EMBO J. (2023) 42:e111901. doi: 10.15252/embj.2022111901 36917141 PMC10233380

[49] Gao L Xu Z Huang Z Tang Y Yang D Huang J . Cpi-613 rewires lipid metabolism to enhance pancreatic cancer apoptosis via the Ampk-Acc signaling. J Exp Clin Cancer Res CR. (2020) 39:73. doi: 10.1186/s13046-020-01579-x 32345326 PMC7187515

[50] Nishi K Suzuki M Yamamoto N Matsumoto A Iwase Y Yamasaki K . Glutamine deprivation enhances acetyl-Coa carboxylase inhibitor-induced death of human pancreatic cancer cells. Anticancer Res. (2018) 38:6683–9. doi: 10.21873/anticanres.13036 30504377

[51] Gradiz R Silva HC Carvalho L Botelho MF Mota-Pinto A . Mia Paca-2 and Panc-1 – pancreas ductal adenocarcinoma cell lines with neuroendocrine differentiation and somatostatin receptors. Sci Rep. (2016) 6:21648. doi: 10.1038/srep21648 26884312 PMC4756684

[52] Daemen A Peterson DA Sahu N McCord R Du X Liu B . Metabolite profiling stratifies pancreatic ductal adenocarcinomas into subtypes with distinct sensitivities to metabolic inhibitors. Proc Natl Acad Sci. (2015) 112:E4410–7. doi: 10.1073/pnas.1501605112 26216984 PMC4538616

[53] Sun YL He W Luo M Zhou Y-H Chang G-L Ren W-Y . Srebp1 regulates tumorigenesis and prognosis of pancreatic cancer through targeting lipid metabolism. Tumor Biol. (2015) 36:4133–41. doi: 10.1007/s13277-015-3047-5 25589463

[54] Svensson RU Parker SJ Eichner LJ Kolar MJ Wallace M Brun SN . Inhibition of acetyl-Coa carboxylase suppresses fatty acid synthesis and tumor growth of non-small cell lung cancer in preclinical models. Nat Med. (2016) 22:1108–19. doi: 10.1038/nm.4181 27643638 PMC5053891

[55] Lally JSV Ghoshal S DePeralta DK Moaven O Wei L Masia R . Inhibition of acetyl-Coa carboxylase (Acc) by phosphorylation or by the liver-specific inhibitor, Nd-654, suppresses lipogenesis and hepatocellular carcinoma. Cell Metab. (2018) 29:174–182.e5. doi: 10.1016/j.cmet.2018.08.020 PMC6643297 30244972

[56] A phase 2, randomized, double-blind, double-dummy, placebo-controlled study evaluating the safety and efficacy of semaglutide, and the fixed-dose combination of cilofexor and firsocostat, alone and in combination, in subjects with compensated cirrhosis (F4) due to nonalcoholic steatohepatitis (Nash) [Internet (2021). Available online at: https://clinicaltrials.gov/study/NCT04971785 (Accessed October 19, 2026).

[57] Matsushita Y Nakagawa H Koike K . Lipid metabolism in oncology: Why it matters, how to research, and how to treat. Cancers. (2021) 13:474. doi: 10.3390/cancers13030474 33530546 PMC7865757

[58] Hao J-W Wang J Guo H Zhao Y-Y Sun H-H Li Y . Cd36 facilitates fatty acid uptake by dynamic palmitoylation-regulated endocytosis. Nat Commun. (2020) 11:4765. doi: 10.1038/s41467-020-18565-8 32958780 PMC7505845

[59] Nath A Li I Roberts LR Chan C . Elevated free fatty acid uptake via Cd36 promotes epithelial-mesenchymal transition in hepatocellular carcinoma. Sci Rep. (2015) 5:14752. doi: 10.1038/srep14752 26424075 PMC4589791

[60] Li Z-R Kang Y . Lipid metabolism fuels cancer's spread. Cell Metab. (2017) 25:228–30. doi: 10.1016/j.cmet.2017.01.016 28178563

[61] Cai Z Li Y Ma M Wang L Wang H Liu M . Adipocytes promote pancreatic cancer migration and invasion through fatty acid metabolic reprogramming. Oncol Rep. (2023) 50:141. doi: 10.3892/or.2023.8578 37264956 PMC10285608

[62] Poenaru RC Milanesi E Niculae AM Dobre A-M Vlăduț C Ciocîrlan M-C . Dysregulation of genes involved in the long-chain fatty acid transport in pancreatic ductal adenocarcinoma. World J Gastrointestinal Oncol. (2025) 17:98409. doi: 10.4251/wjgo.v17.i1.98409 39817147 PMC11664611

[63] Wang Y Zhang S Song Y An L Zhao Z-T Zhou Y . Multi-omic profiling reveals Smad4 drives linoleic acid-arachidonic acid metabolism to mediate Pdac radiosensitivity via the Slc27a3/Fads2 axis. Biochem Biophys Res Commun. (2026) 817:153758. doi: 10.1016/j.bbrc.2026.153758 41990451

[64] Commisso C Davidson SM Soydaner-Azeloglu RG Parker SJ Kamphorst JJ Hackett SR . Macropinocytosis of protein is an amino acid supply route in Ras-transformed cells. Nature. (2013) 497:633–7. doi: 10.1038/nature12138 23665962 PMC3810415

[65] Yao W-T Rose JL Wang W Seth S Jiang H Taguchi A . Syndecan1 is a critical mediator of macropinocytosis in pancreatic cancer. Nature. (2019) 568:410–4. doi: 10.1038/s41586-019-1062-1 30918400 PMC6661074

[66] Hobbs GA Baker NM Miermont AM Thurman RD Pierobon M Tran TH . Atypical Krasg12r mutant is impaired in Pi3k signaling and macropinocytosis in pancreatic cancer. Cancer Discov. (2019) 10:104–23. doi: 10.1158/2159-8290.CD-19-1006 PMC6954322 31649109

[67] Davidson SM Jonas O Keibler MA Hou HW Luengo A Mayers JR . Direct evidence for cancer cell-autonomous extracellular protein catabolism in pancreatic tumors. Nat Med. (2016) 23:235–41. doi: 10.1038/nm.4256 28024083 PMC5407288

[68] Kamphorst JJ Nofal M Commisso C Hackett SR Lu W Grabocka E . Human pancreatic cancer tumors are nutrient poor and tumor cells actively scavenge extracellular protein. Cancer Res. (2015) 75:544–53. doi: 10.1158/0008-5472.can-14-2211 25644265 PMC4316379

[69] Skrypek K Balog S Eriguchi Y Asahina K . Inhibition of stearoyl-Coa desaturase induces the unfolded protein response in pancreatic tumors and suppresses their growth. Pancreas. (2021) 50:219–26. doi: 10.1097/mpa.0000000000001737 33565799 PMC7880535

[70] Ntambi JM Miyazaki M . Regulation of stearoyl-Coa desaturases and role in metabolism. Prog Lipid Res. (2004) 43:91–104. doi: 10.1016/s0163-7827(03)00039-0 14654089

[71] Miyazaki M Ntambi JM . Role of stearoyl-coenzyme a desaturase in lipid metabolism. Prostaglandins Leukotrienes Essential Fatty Acids. (2003) 68:113–21. doi: 10.1016/s0952-3278(02)00261-2 12538075

[72] Piccolis M Bond LM Kampmann M Pulimeno P Chitraju C Jayson C . Probing the global cellular responses to lipotoxicity caused by saturated fatty acids. Mol Cell. (2019) 74:32–44.e8. doi: 10.1016/j.molcel.2019.01.036 30846318 PMC7696670

[73] Kubota CS Espenshade PJ . Targeting stearoyl-Coa desaturase in solid tumors. Cancer Res. (2022) 82:1682–8. doi: 10.1158/0008-5472.can-21-4044 35294526 PMC9064960

[74] Lin S Tan S Peng Y Tulamaiti A Du W Ding K . Histone serotonylation promotes pancreatic cancer development via lipid metabolism remodeling. Nat Commun. (2025) 16:5947. doi: 10.1038/s41467-025-61197-z 40593695 PMC12217961

[75] Liu Y Wang Y Lin Z Kang R Tang D Liu J . Slc25a22 as a key mitochondrial transporter against ferroptosis by producing glutathione and monounsaturated fatty acids. Antioxid Redox Signal. (2023) 39:166–85. doi: 10.1089/ars.2022.0203 37051693 PMC10620438

[76] Zhang Z Cai X Gong Y Gong A Liao X Gao J . Srebp1-Scd1 enhanced Mufas biosynthesis drives nutrient deprived pancreatic cancer cell ferroptosis resistance. J Cancer. (2025) 16:3960–71. doi: 10.7150/jca.114356 41049011 PMC12491149

[77] Zhang X Zhu B Yan J Chen X Wu D Wang Z . Matrix stiffness boosts Pdac chemoresistance via Scd1-dependent lipid metabolic reprogramming. Regener Biomater. (2025) 12:rbaf056. doi: 10.1093/rb/rbaf056 40741620 PMC12308179

[78] Cui D Yu X Guan Q Shen Y Liao J Liu Y . Cholesterol metabolism: Molecular mechanisms, biological functions, diseases, and therapeutic targets. Mol BioMed. (2025) 6:72. doi: 10.1186/s43556-025-00321-3 41062796 PMC12508344

[79] Swanson KM Hohl RJ . Anti-cancer therapy: Targeting the mevalonate pathway. Curr Cancer Drug Targets. (2006) 6:15–37. doi: 10.2174/156800906775471743 16475974

[80] Istvan ES Palnitkar M Buchanan SK Deisenhofer J . Crystal structure of the catalytic portion of human Hmg‐Coa reductase: Insights into regulation of activity and catalysis. EMBO J. (2000) 19:819–30. doi: 10.1093/emboj/19.5.819 10698924 PMC305622

[81] Madison BB . Srebp2: A master regulator of sterol and fatty acid synthesis1. J Lipid Res. (2016) 57:333–5. doi: 10.1194/jlr.c066712 26798145 PMC4766982

[82] Sever N Song B-L Yabe D Goldstein JL Brown MS DeBose-Boyd RA . Insig-dependent ubiquitination and degradation of mammalian 3-hydroxy-3-methylglutaryl-Coa reductase stimulated by sterols and geranylgeraniol*. J Biol Chem. (2003) 278:52479–90. doi: 10.1074/jbc.m310053200 14563840

[83] Chen L Ma M-Y Sun M-S Jiang L Zhao X-T Fang X-X . Endogenous sterol intermediates of the mevalonate pathway regulate Hmgcr degradation and Srebp-2 processing[s. J Lipid Res. (2019) 60:1765–75. doi: 10.1194/jlr.ra119000201 31455613 PMC6795084

[84] Carrer A Trefely S Zhao S Campbell SL Norgard RJ Schultz KC . Acetyl-Coa metabolism supports multistep pancreatic tumorigenesis. Cancer Discov. (2019) 9:416–35. doi: 10.1158/2159-8290.Cd-18-0567 30626590 PMC6643997

[85] Ishikawa S Nagai Y Masuda T Koga Y Nakamura T Imamura Y . The role of oxysterol binding protein‐related protein 5 in pancreatic cancer. Cancer Sci. (2010) 101:898–905. doi: 10.1111/j.1349-7006.2009.01475.x PMC11159417 20128820

[86] Sun R Qin H Zhang W Guo X Teng Y Xu M . The Trib1–Pparγ axis regulates cholesterol metabolism in pancreatic ductal adenocarcinoma. Ann N Y Acad Sci. (2026) 1557:e70224. doi: 10.1111/nyas.70224 41774091

[87] LiF YangM SunX ChenL ZhengD WeiZ . Hoxc9 enhances cholesterol metabolism and malignancy in pancreatic ductal adenocarcinoma through Itga10/Fak/Pi3k/Creb-dependent Hmgcr activation. J Gastroenterol. (2026). doi: 10.1007/s00535-026-02457-742283762

[88] Shimada K Skouta R Kaplan A Yang WS Hayano M Dixon SJ . Global survey of cell death mechanisms reveals metabolic regulation of ferroptosis. Nat Chem Biol. (2016) 12:497–503. doi: 10.1038/nchembio.2079 27159577 PMC4920070

[89] Bentinger M Tekle M Dallner G . Coenzyme Q--biosynthesis and functions. Biochem Biophys Res Commun. (2010) 396:74–9. doi: 10.1016/j.bbrc.2010.02.147 20494114

[90] Bersuker K Hendricks JM Li Z Magtanong L Ford B Tang PH . The Coq oxidoreductase Fsp1 acts in parallel to Gpx4 to inhibit ferroptosis. Nature. (2019) 575:688–92. doi: 10.1038/s41586-019-1705-2 31634900 PMC6883167

[91] Doll S Freitas FP Shah R Aldrovandi M Silva MC Ingold I . Fsp1 is a glutathione-independent ferroptosis suppressor. Nature. (2019) 575:693–8. doi: 10.1038/s41586-019-1707-0 31634899

[92] Nielsen SF Nordestgaard BG Bojesen SE . Statin use and reduced cancer-related mortality. N Engl J Med. (2012) 367:1792–802. doi: 10.1056/nejmoa1201735 23134381

[93] Longo J van Leeuwen JE Elbaz M Branchard E Penn LZ . Statins as anticancer agents in the era of precision medicine. Clin Cancer Res. (2020) 26:5791–800. doi: 10.1158/1078-0432.ccr-20-1967 32887721

[94] Uemura N Hayashi H Liu Z Matsumura K Ogata Y Yasuda N . Statins exert anti-growth effects by suppressing Yap/Taz expressions via Jnk signal activation and eliminate the immune suppression by downregulating Pd-L1 expression in pancreatic cancer. Am J Cancer Res. (2023) 13:2041–54. PMC10244110 37293171

[95] Hamada T Khalaf N Yuan C Morales-Oyarvide V Babic A Nowak JA . Pre-diagnosis use of statins associates with increased survival times of patients with pancreatic cancer. Clin Gastroenterol Hepatol Off Clin Pract J Am Gastroenterological Assoc. (2018) 16:1300–1306.e3. doi: 10.1016/j.cgh.2018.02.022 29474971 PMC6056316

[96] Wu BU Chang JI Jeon CY Pandol SJ Huang BZ Ngor EW . Impact of statin use on survival in patients undergoing resection for early-stage pancreatic cancer. Am J Gastroenterol. (2015) 110:1233–9. doi: 10.1038/ajg.2015.217 26195180 PMC4877304

[97] Hamada T Khalaf N Yuan C Babic A Morales-Oyarvide V Qian ZR . Statin use and pancreatic cancer risk in two prospective cohort studies. J Gastroenterol. (2018) 53:959–66. doi: 10.1007/s00535-018-1430-x 29362938 PMC7609961

[98] Simon MS Desai PM Wallace RB Wu C Howard BV Martin LW . Prospective analysis of association between statins and pancreatic cancer risk in the Women’s Health Initiative. Cancer Causes Control CCC. (2016) 27:415–23. doi: 10.1007/s10552-016-0717-6 26857832 PMC6849382

[99] Jacobs EJ Newton CC Thun MJ Gapstur SM . Long-term use of cholesterol-lowering drugs and cancer incidence in a large United States cohort. Cancer Res. (2010) 71:1763–71. doi: 10.1158/1940-6207.prev-10-b95 21343395

[100] Kawashiri T Tokunaga A Kobayashi D Shimazoe T . Anti-tumor activities of 3-hydroxy-3-methylglutaryl coenzyme a (Hmg-Coa) reductase inhibitors and bisphosphonates in pancreatic cell lines which show poor responses to gemcitabine. Biol Pharm Bull. (2020) 43:49–52. doi: 10.1248/bpb.b19-00435 31902931

[101] Padyana AK Gross S Jin L Cianchetta G Narayanaswamy R Wang F . Structure and inhibition mechanism of the catalytic domain of human squalene epoxidase. Nat Commun. (2019) 10:97. doi: 10.1038/s41467-018-07928-x 30626872 PMC6327030

[102] Gill S Stevenson J Kristiana I Brown AJ . Cholesterol-dependent degradation of squalene monooxygenase, a control point in cholesterol synthesis beyond Hmg-Coa reductase. Cell Metab. (2011) 13:260–73. doi: 10.1016/j.cmet.2011.01.015 21356516

[103] Zelcer N Sharpe LJ Loregger A Kristiana I Cook ECL Phan LE . The E3 ubiquitin ligase March6 degrades squalene monooxygenase and affects 3-hydroxy-3-methyl-glutaryl coenzyme a reductase and the cholesterol synthesis pathway. Mol Cell Biol. (2014) 34:1262–70. doi: 10.1128/mcb.01140-13 24449766 PMC3993563

[104] Zhao F-Q Huang Y Zhang Y Li X Chen K Long Y . Sqle inhibition suppresses the development of pancreatic ductal adenocarcinoma and enhances its sensitivity to chemotherapeutic agents *in vitro*. Mol Biol Rep. (2022) 49:6613–21. doi: 10.21203/rs.3.rs-1330088/v1 35552960

[105] Wang S Dong L Ma L Yang S Zheng Y Zhang J . Sqle facilitates the pancreatic cancer progression via the Lncrna‐Ttn‐As1/Mir‐133b/Sqle axis. J Cell Mol Med. (2022) 26:3636–47. doi: 10.1111/jcmm.17347 PMC9258714 35638462

[106] Pan J Liu R Lu W Peng H Yang J Zhang Q . Sqle-catalyzed squalene metabolism promotes mitochondrial biogenesis and tumor development in K-Ras-driven cancer. Cancer Lett. (2025) 616:217586. doi: 10.1016/j.canlet.2025.217586 40015662

[107] Pan J Liang H Zhou L Lu W Huo B Liu R . Sqle-mediated squalene metabolism promotes tumor immune evasion in pancreatic cancer. Front Immunol. (2024) 15. doi: 10.3389/fimmu.2024.1512981 39763673 PMC11701373

[108] Souchek JJ Baine MJ Lin C Rachagani S Gupta S Kaur S . Unbiased analysis of pancreatic cancer radiation resistance reveals cholesterol biosynthesis as a novel target for radiosensitisation. Br J Cancer. (2014) 111:1139–49. doi: 10.1038/bjc.2014.385 25025965 PMC4453840

[109] He Y Zhu Y Wang W Yi Y Wang Z Zhao C . Clinical efficacy and chemoresistance analysis of precision neoadjuvant chemotherapy for borderline resectable pancreatic cancer: A prospective, single-arm pilot study. Int J Surg (London England). (2025) 111:3269–80. doi: 10.1097/js9.0000000000002342 40146255 PMC12165568

[110] Wei W Wang H Shi DD Ai L Liu H . Icaritin disrupts cholesterol biosynthesis and induce cell death in pancreatic carcinoma: A transcriptomic and lipidomic analysis. Chin J Integr Med. (2026) 32:442–50. doi: 10.1007/s11655-026-4236-6 41920258

[111] van Loon NM van Wouw SAE Ottenhoff R Nelson JK Kingma J Scheij S . Regulation of intestinal Ldlr by the Lxr-Idol axis. Atherosclerosis. (2020) 315:1–9. doi: 10.1016/j.atherosclerosis.2020.10.898 33190106

[112] Li F Zhang H . Lysosomal acid lipase in lipid metabolism and beyond. Arteriosclerosis Thrombosis Vasc Biol. (2019) 39:850–6. doi: 10.1161/atvbaha.119.312136 30866656 PMC6482091

[113] Frolov AA Zielinski SE Crowley JR Dudley-Rucker N Schaffer JE Ory DS . Npc1 and Npc2 regulate cellular cholesterol homeostasis through generation of low density lipoprotein cholesterol-derived oxysterols*. J Biol Chem. (2003) 278:25517–25. doi: 10.1074/jbc.m302588200 12719428

[114] Guillaumond F Bidaut G Ouaissi M Servais S Gouirand V Olivares O . Cholesterol uptake disruption, in association with chemotherapy, is a promising combined metabolic therapy for pancreatic adenocarcinoma. Proc Natl Acad Sci. (2015) 112:2473–8. doi: 10.1073/pnas.1421601112 25675507 PMC4345573

[115] Xiao J Dong L Liu S Meng F-H Xie C Lu X-Y . Bile acids-mediated intracellular cholesterol transport promotes intestinal cholesterol absorption and Npc1l1 recycling. Nat Commun. (2023) 14:6469. doi: 10.1038/s41467-023-42179-5 37833289 PMC10575946

[116] Huang C-S Yu X-C Fordstrom P Choi K Chung BC Roh SH . Cryo-Em structures of Npc1l1 reveal mechanisms of cholesterol transport and ezetimibe inhibition. Sci Adv. (2020) 6:eabb1989. doi: 10.1126/sciadv.abb1989 32596471 PMC7304964

[117] Zi R Shen K Zheng P Su X Yang Y Chen Y . Npc1l1 on pancreatic adenocarcinoma cell functions as a two-pronged checkpoint against antitumor activity. Innovation. (2025) 6:100783. doi: 10.1016/j.xinn.2024.100783 40098667 PMC11910884

[118] Brown MS Radhakrishnan A Goldstein JL . Retrospective on cholesterol homeostasis: The central role of Scap. Annu Rev Biochem. (2017) 87:783–807. doi: 10.1146/annurev-biochem-062917-011852 PMC5828883 28841344

[119] Goldstein JL DeBose-Boyd RA Brown MS . Protein sensors for membrane sterols. Cell. (2006) 124:35–46. doi: 10.1016/j.cell.2005.12.022 16413480

[120] Radhakrishnan A Goldstein JL McDonald JG Brown MS . Switch-like control of Srebp-2 transport triggered by small changes in Er cholesterol: A delicate balance. Cell Metab. (2008) 8:512–21. doi: 10.1016/j.cmet.2008.10.008 19041766 PMC2652870

[121] Infante RE Radhakrishnan A . Continuous transport of a small fraction of plasma membrane cholesterol to endoplasmic reticulum regulates total cellular cholesterol. eLife. (2017) 6:e25466. doi: 10.7554/elife.25466 28414269 PMC5433840

[122] Garcia BM Melchinger P Medeiros TC Hendrix S Prabhu K Corrado M . Glutamine sensing licenses cholesterol synthesis. EMBO J. (2024) 43:5837–56. doi: 10.1038/s44318-024-00269-0 39433901 PMC11612431

[123] Sussman JH Kim N Kemp SB Traum D Katsuda T Kahn BM . Multiplexed imaging mass cytometry analysis characterizes the vascular niche in pancreatic cancer. Cancer Res. (2024) 84:2364–76. doi: 10.1158/0008-5472.can-23-2352 38695869 PMC11250934

[124] Chitty JL Yam M Perryman L Parker AL Skhinas JN Setargew YFI . A first-in-class pan-lysyl oxidase inhibitor impairs stromal remodeling and enhances gemcitabine response and survival in pancreatic cancer. Nat Cancer. (2023) 4:1326–44. doi: 10.1038/s43018-023-00614-y 37640930 PMC10518255

[125] Sullivan MR Danai LV Lewis CA Chan SH Gui DY Kunchok T . Quantification of microenvironmental metabolites in murine cancers reveals determinants of tumor nutrient availability. eLife. (2019) 8:e44235. doi: 10.7554/eLife.44235 30990168 PMC6510537

[126] Apiz Saab JJ Dzierozynski LN Jonker PB AminiTabrizi R Shah H Menjivar RE . Pancreatic tumors exhibit myeloid-driven amino acid stress and upregulate arginine biosynthesis. eLife. (2023) 12:e81289. doi: 10.7554/eLife.81289 PMC10260022 37254839

[127] Rogers MA Liu J Song B Li B-L Chang CCY Chang T-Y . Acyl-Coa:Cholesterol acyltransferases (Acats/Soats): Enzymes with multiple sterols as substrates and as activators. J Steroid Biochem Mol Biol. (2014) 151:102–7. doi: 10.1016/j.jsbmb.2014.09.008 PMC4851438 25218443

[128] Oni TE Biffi G Baker LA Hao Y Tonelli C Somerville TDD . Soat1 promotes mevalonate pathway dependency in pancreatic cancer. J Exp Med. (2020) 217:e20192389. doi: 10.1084/jem.20192389 32633781 PMC7478739

[129] Li J Gu D Lee S-Y Song B Bandyopadhyay S Chen S . Abrogating cholesterol esterification suppresses growth and metastasis of pancreatic cancer. Oncogene. (2016) 35:6378–88. doi: 10.1038/onc.2016.168 27132508 PMC5093084

[130] Maki R Iwagami Y Kobayashi S Sasaki K Yamada D Tomimaru Y . Efficacy of Soat1 inhibitors against pancreatic cancer with Tp53 mutations. Ann Surg Oncol. (2025) 32:7025–37. doi: 10.1245/s10434-025-17482-8 40445510

[131] Lee S-Y Li J Tai JN Ratliff TL Park K Cheng JX . Avasimibe encapsulated in human serum albumin blocks cholesterol esterification for selective cancer treatment. ACS Nano. (2015) 9:2420–32. doi: 10.1021/nn504025a 25662106 PMC5909415

[132] Li J Qu XJ Tian J Zhang J-T Cheng JX . Cholesterol esterification inhibition and gemcitabine synergistically suppress pancreatic ductal adenocarcinoma proliferation. PloS One. (2018) 13:e0193318. doi: 10.1371/journal.pone.0193318 29489864 PMC5831104

[133] Cruz ALS de A. Barreto E Fazolini NPB Viola JPB Bozza PT . Lipid droplets: Platforms with multiple functions in cancer hallmarks. Cell Death Dis. (2020) 11:105. doi: 10.1038/s41419-020-2297-3 32029741 PMC7005265

[134] Grachan JJ Kery M Giaccia AJ Denko NC Papandreou I . Lipid droplet storage promotes murine pancreatic tumor growth. Oncol Rep. (2021) 45:21. doi: 10.3892/or.2021.7972 33649859 PMC8889526

[135] Gotvaldová K Špačková J Novotný J Baslarová K Ježek P Rossmeislová L . Bcaa metabolism in pancreatic cancer affects lipid balance by regulating fatty acid import into mitochondria. Cancer Metab. (2024) 12:10. doi: 10.1186/s40170-024-00335-5 PMC10967191 38532464

[136] Kuramoto K Yamamoto M Suzuki S Togashi K Sanomachi T Kitanaka C . Inhibition of the lipid droplet–peroxisome proliferator-activated receptor A axis suppresses cancer stem cell properties. Genes. (2021) 12:99. doi: 10.3390/genes12010099 33466690 PMC7828779

[137] Petan T Jarc E Jusović M . Lipid droplets in cancer: Guardians of fat in a stressful world. Molecules A J Synthetic Chem Natural Product Chem. (2018) 23:1941. doi: 10.3390/molecules23081941 30081476 PMC6222695

[138] Lu G-D Yang H-A Zhou J Tamilarasi J Yan B Lim YC . Ccaat/enhancer binding protein a predicts poorer prognosis and prevents energy starvation–induced cell death in hepatocellular carcinoma. Hepatol (Baltimore Md). (2015) 61:965–78. doi: 10.1002/hep.27593 25363290 PMC4365685

[139] Mukhopadhyay S Schlaepfer IR Bergman BC Panda PK Praharaj PP Naik PP . Atg14 facilitated lipophagy in cancer cells induce Er stress mediated mitoptosis through a Ros dependent pathway. Free Radical Biol Med. (2017) 104:199–213. doi: 10.1016/j.freeradbiomed.2017.01.007 28069524

[140] Duan Z Ma X Yu F Zhang J Hua R Liu W . Osbpl10 drives lipophagy-mediated lipid mobilization to promote pancreatic ductal adenocarcinoma progression. Int J Biol Sci. (2026) 22:3769–87. doi: 10.7150/ijbs.125552 42003919 PMC13086084

[141] Saliakoura M Sebastiano MR Nikdima I Pozzato C Konstantinidou G . Restriction of extracellular lipids renders pancreatic cancer dependent on autophagy. J Exp Clin Cancer Res CR. (2022) 41:16. doi: 10.1186/s13046-021-02231-y 34998392 PMC8742413

[142] Zhang Z-H Aoki H Umezawa K Kranrod JW Miyazaki N Oshima T . Potential role of lipophagy impairment for anticancer effects of glycolysis-suppressed pancreatic ductal adenocarcinoma cells. Cell Death Discov. (2024) 10:166. doi: 10.1038/s41420-024-01933-4 38580661 PMC10997792

[143] Rebelo R Xavier CPR Giovannetti E Vasconcelos MH . Fibroblasts in pancreatic cancer: Molecular and clinical perspectives. Trends Mol Med. (2023) 29:439–53. doi: 10.1016/j.molmed.2023.03.002 37100646

[144] Sancho P Burgos-Ramos E Tavera A Bou Kheir T Jagušt P Schoenhals M . Myc/Pgc-1α balance determines the metabolic phenotype and plasticity of pancreatic cancer stem cells. Cell Metab. (2015) 22:590–605. doi: 10.1016/j.cmet.2015.08.015 26365176

[145] Manzo T Prentice BM Anderson KG Raman A Schalck A Codreanu GS . Accumulation of long-chain fatty acids in the tumor microenvironment drives dysfunction in intrapancreatic Cd8+ T cells. J Exp Med. (2020) 217:e20191920. doi: 10.1084/jem.20191920 32491160 PMC7398173

[146] Niu N Shen X Wang Z Chen Y Weng Y Yu F . Tumor cell-intrinsic epigenetic dysregulation shapes cancer-associated fibroblasts heterogeneity to metabolically support pancreatic cancer. Cancer Cell. (2024) 42:869–884.e9. doi: 10.1016/j.ccell.2024.03.005 38579725

[147] Okumura T Ohuchida K Sada M Abe T Endo S Koikawa K . Extra-pancreatic invasion induces lipolytic and fibrotic changes in the adipose microenvironment, with released fatty acids enhancing the invasiveness of pancreatic cancer cells. Oncotarget. (2017) 8:18280–95. doi: 10.18632/oncotarget.15430 28407685 PMC5392327

[148] Liotta LA Kohn EC . The microenvironment of the tumour-host interface. Nature. (2001) 411:375–9. doi: 10.1038/35077241 11357145

[149] Fiori ME Di Franco S Villanova L Bianca P Stassi G De Maria R . Cancer-associated fibroblasts as abettors of tumor progression at the crossroads of Emt and therapy resistance. Mol Cancer. (2019) 18:70. doi: 10.1186/s12943-019-0994-2 30927908 PMC6441236

[150] Orimo A Gupta PB Sgroi DC Arenzana-Seisdedos F Delaunay T Naeem R . Stromal fibroblasts present in invasive human breast carcinomas promote tumor growth and angiogenesis through elevated Sdf-1/Cxcl12 secretion. Cell. (2005) 121:335–48. doi: 10.1016/j.cell.2005.02.034 15882617

[151] Ying H Kimmelman AC Lyssiotis CA Hua S Chu GC Fletcher-Sananikone E . Oncogenic kras maintains pancreatic tumors through regulation of anabolic glucose metabolism. Cell. (2012) 149:656–70. doi: 10.1016/j.cell.2012.01.058 22541435 PMC3472002

[152] Auciello FR Bulusu V Oon C Tait-Mulder J Berry M Bhattacharyya S . A stromal lysolipid-autotaxin signaling axis promotes pancreatic tumor progression. Cancer Discov. (2019) 9:617–27. doi: 10.1158/2159-8290.Cd-18-1212 30837243 PMC6497553

[153] Wang Y Liang Y-Y Xu H-Y Zhang X Mao T-B Cui J . Single-cell analysis of pancreatic ductal adenocarcinoma identifies a novel fibroblast subtype associated with poor prognosis but better immunotherapy response. Cell Discov. (2021) 7:36. doi: 10.1038/s41421-021-00271-4 34035226 PMC8149399

[154] Hu B-Y Wu CC Mao H Gu H Dong H Yan J . Subpopulations of cancer-associated fibroblasts link the prognosis and metabolic features of pancreatic ductal adenocarcinoma. Ann Trans Med. (2022) 10:262. doi: 10.21037/atm-22-407 35402584 PMC8987890

[155] Cho M Koh E Kim YY Kim S-J Pack C-G Jun E . Molecular profiling of inflammatory and myofibroblast cancer-associated fibroblast subtypes derived from human pancreatic stellate cells using machine learning-based label-free raman spectroscopy. Biomater Res. (2025) 29:0292. doi: 10.34133/bmr.0292 41376815 PMC12686345

[156] Zhang T Ren Y Yang P Wang J Zhou H . Cancer-associated fibroblasts in pancreatic ductal adenocarcinoma. Cell Death Dis. (2022) 13:897. doi: 10.1038/s41419-022-05351-1 36284087 PMC9596464

[157] Sherman MH Yu RT Engle DD Ding N Atkins AR Tiriac H . Vitamin d receptor-mediated stromal reprogramming suppresses pancreatitis and enhances pancreatic cancer therapy. Cell. (2014) 159:80–93. doi: 10.1016/j.cell.2014.08.007 25259922 PMC4177038

[158] Kim N Yoo W Lee J Kim H Lee H Kim YS . Formation of vitamin a lipid droplets in pancreatic stellate cells requires albumin. Gut. (2009) 58:1382–90. doi: 10.1136/gut.2008.170233 19293176

[159] Saison-Ridinger M DelGiorno KE Zhang T Kraus A French R Jaquish D . Reprogramming pancreatic stellate cells via p53 activation: a putative target for pancreatic cancer therapy. PloS One. (2017) 12:e0189051. doi: 10.1371/journal.pone.0189051 29211796 PMC5718507

[160] Schnittert J Heinrich MA Kuninty PR Storm G Prakash J . Reprogramming tumor stroma using an endogenous lipid lipoxin a4 to treat pancreatic cancer. Cancer Lett. (2018) 420:247–58. doi: 10.1016/j.canlet.2018.01.072 29408203

[161] Bhattacharyya S Oon C Diaz L Sandborg H Stempinski ES Saoi M . Autotaxin-lysolipid signaling suppresses a ccl11-eosinophil axis to promote pancreatic cancer progression. Nat Cancer. (2024) 5:283–98. doi: 10.1038/s43018-023-00703-y 38195933 PMC10899115

[162] Bulle A Lim KH . Beyond just a tight fortress: contribution of stroma to epithelial-mesenchymal transition in pancreatic cancer. Signal Transduct Target Ther. (2020) 5:249. doi: 10.1038/s41392-020-00341-1 33122631 PMC7596088

[163] Young RM Ackerman D Quinn ZL Mancuso A Gruber M Liu L . Dysregulated mtorc1 renders cells critically dependent on desaturated lipids for survival under tumor-like stress. Genes Dev. (2013) 27:1115–31. doi: 10.1101/gad.198630.112 23699409 PMC3672646

[164] Han X Kim LC Lesner NP Cai X Van Le TN Simon MC . Glutaminase inhibition ameliorates cancer-associated fibroblast lipid support of pancreatic cancer cell growth. Cancer Metab. (2025) 13:38. doi: 10.1186/s40170-025-00389-z 40835956 PMC12366226

[165] Qi R Bai Y Li K Liu N Xu Y Dal E . Cancer-associated fibroblasts suppress ferroptosis and induce gemcitabine resistance in pancreatic cancer cells by secreting exosome-derived acsl4-targeting mirnas. Drug Resist Update. (2023) 68:100960. doi: 10.1016/j.drup.2023.100960 37003125

[166] Zhang M Di Martino JS Bowman RL Campbell NR Baksh SC Simon-Vermot T . Adipocyte-derived lipids mediate melanoma progression via fatp proteins. Cancer Discov. (2018) 8:1006–25. doi: 10.1158/2159-8290.Cd-17-1371 29903879 PMC6192670

[167] Li F Simon MC . Cancer cells don't live alone: metabolic communication within tumor microenvironments. Dev Cell. (2020) 54:183–95. doi: 10.1016/j.devcel.2020.06.018 32640203 PMC7375918

[168] Yu W Lei Q Yang L Qin G Liu S Wang D . Contradictory roles of lipid metabolism in immune response within the tumor microenvironment. J Hematol Oncol. (2021) 14:187. doi: 10.1186/s13045-021-01200-4 34742349 PMC8572421

[169] Clark CE Hingorani SR Mick R Combs C Tuveson DA Vonderheide RH . Dynamics of the immune reaction to pancreatic cancer from inception to invasion. Cancer Res. (2007) 67:9518–27. doi: 10.1158/0008-5472.Can-07-0175 17909062

[170] DeBerardinis RJ Chandel NS . Fundamentals of cancer metabolism. Sci Adv. (2016) 2:e1600200. doi: 10.1126/sciadv.1600200 27386546 PMC4928883

[171] Jin R Neufeld L McGaha TL . Linking macrophage metabolism to function in the tumor microenvironment. Nat Cancer. (2025) 6:239–52. doi: 10.1038/s43018-025-00909-2 39962208

[172] Imianowski CJ Chen Q Workman CJ Vignali DAA . Regulatory t cells in the tumour microenvironment. Nat Rev Cancer. (2025) 25:703–22. doi: 10.1038/s41568-025-00832-9 40494998

[173] Martin-Perez M Urdiroz-Urricelqui U Bigas C Benitah SA . The role of lipids in cancer progression and metastasis. Cell Metab. (2022) 34:1675–99. doi: 10.1016/j.cmet.2022.09.023 36261043

[174] Ping Y Shan J Qin H Li F Qu J Guo R . Pd-1 signaling limits expression of phospholipid phosphatase 1 and promotes intratumoral cd8(+) t cell ferroptosis. Immunity. (2024) 57:2122–2139.e9. doi: 10.1016/j.immuni.2024.08.003 39208806

[175] Ma X Bi E Lu Y Su P Huang C Liu L . Cholesterol induces cd8(+) t cell exhaustion in the tumor microenvironment. Cell Metab. (2019) 30:143–156.e5. doi: 10.1016/j.cmet.2019.04.002 31031094 PMC7061417

[176] Yang P Qin H Li Y Xiao A Zheng E Zeng H . Cd36-mediated metabolic crosstalk between tumor cells and macrophages affects liver metastasis. Nat Commun. (2022) 13:5782. doi: 10.1038/s41467-022-33349-y 36184646 PMC9527239

[177] Su P Wang Q Bi E Ma X Liu L Yang M . Enhanced lipid accumulation and metabolism are required for the differentiation and activation of tumor-associated macrophages. Cancer Res. (2020) 80:1438–50. doi: 10.1158/0008-5472.Can-19-2994 32015091 PMC7127942

[178] Morrison AH Byrne KT Vonderheide RH . Immunotherapy and prevention of pancreatic cancer. Trends Cancer. (2018) 4:418–28. doi: 10.1016/j.trecan.2018.04.001 29860986 PMC6028935

[179] Stromnes IM Hulbert A Pierce RH Greenberg PD Hingorani SR . T-cell localization, activation, and clonal expansion in human pancreatic ductal adenocarcinoma. Cancer Immunol Res. (2017) 5:978–91. doi: 10.1158/2326-6066.cir-16-0322 29066497 PMC5802342

[180] Wu J Qian P Han Y Xu C Xia M Zhan P . Glp1 alleviates oleic acid-propelled lipocalin-2 generation by tumor-infiltrating cd8(+) t cells to reduce polymorphonuclear mdsc recruitment and enhances viral immunotherapy in pancreatic cancer. Cell Mol Immunol. (2025) 22:282–99. doi: 10.1038/s41423-025-01260-3 39910336 PMC11868399

[181] McBrearty N Cho C Chen J Zahedi F Peck AR Radaelli E . Tumor-suppressive and immune-stimulating roles of cholesterol 25-hydroxylase in pancreatic cancer cells. Mol Cancer Res. (2023) 21:228–39. doi: 10.1158/1541-7786.Mcr-22-0602 36378658 PMC9992122

[182] Wu B Shen W Wang X Wang J Zhong Z Zhou Z . Plasma lipid levels are associated with the cd8+ t-cell infiltration and prognosis of patients with pancreatic cancer. Cancer Med. (2023) 12:14138–48. doi: 10.1002/cam4.6080 37184113 PMC10358239

[183] Wang H Franco F Tsui YC Xie X Trefny MP Zappasodi R . Cd36-mediated metabolic adaptation supports regulatory t cell survival and function in tumors. Nat Immunol. (2020) 21:298–308. doi: 10.1038/s41590-019-0589-5 32066953 PMC7043937

[184] Kumagai S Togashi Y Sakai C Kawazoe A Kawazu M Ueno T . An oncogenic alteration creates a microenvironment that promotes tumor progression by conferring a metabolic advantage to regulatory t cells. Immunity. (2020) 53:187–203.e8. doi: 10.1016/j.immuni.2020.06.016 32640259

[185] Nakajima T Kanno T Ueda Y Miyako K Endo T Yoshida S . Fatty acid metabolism constrains th9 cell differentiation and antitumor immunity via the modulation of retinoic acid receptor signaling. Cell Mol Immunol. (2024) 21:1266–81. doi: 10.1038/s41423-024-01209-y 39187636 PMC11528006

[186] Jiang H Hegde S Knolhoff BL Zhu Y Herndon JM Meyer MA . Targeting focal adhesion kinase renders pancreatic cancers responsive to checkpoint immunotherapy. Nat Med. (2016) 22:851–60. doi: 10.1038/nm.4123 27376576 PMC4935930

[187] Kemp SB Carpenter ES Steele NG Donahue KL Nwosu ZC Pacheco A . Apolipoprotein e promotes immune suppression in pancreatic cancer through nf-κb-mediated production of cxcl1. Cancer Res. (2021) 81:4305–18. doi: 10.1158/0008-5472.Can-20-3929 34049975 PMC8445065

[188] Yang J Liu S Li Y Fan Z Meng Y Zhou B . Fabp4 in macrophages facilitates obesity-associated pancreatic cancer progression via the nlrp3/il-1β axis. Cancer Lett. (2023) 575:216403. doi: 10.1016/j.canlet.2023.216403 37741433

[189] Caronni N La Terza F Vittoria FM Barbiera G Mezzanzanica L Cuzzola V . Il-1β(+) macrophages fuel pathogenic inflammation in pancreatic cancer. Nature. (2023) 623:415–22. doi: 10.1038/s41586-023-06685-2 37914939

[190] Zhan T Huang M Chen M Chen W Wang Y Chen X . The rassf1c-hif-1α axis drives macrophage lipid metabolism to promote pancreatic cancer. Cell Death Dis. (2026) 17:430. doi: 10.1038/s41419-026-08609-0 41912489 PMC13156295

[191] Xu S Peng X Wang Z Le C Wu X Zeng Z . Fabp7-mediated lipid-laden macrophages drive the formation of pre-metastatic niche and liver metastasis. Int J Biol Sci. (2025) 21:4388–409. doi: 10.7150/ijbs.110750 40765822 PMC12320231

[192] Dai E Han L Liu J Xie Y Kroemer G Klionsky DJ . Autophagy-dependent ferroptosis drives tumor-associated macrophage polarization via release and uptake of oncogenic kras protein. Autophagy. (2020) 16:2069–83. doi: 10.1080/15548627.2020.1714209 31920150 PMC7595620

[193] Zhan T Zou Y Han Z Tian X Chen M Liu J . Single-cell sequencing combined with spatial transcriptomics reveals that the irf7 gene in m1 macrophages inhibits the occurrence of pancreatic cancer by regulating lipid metabolism-related mechanisms. Clin Transl Med. (2024) 14:e1799. doi: 10.1002/ctm2.1799 39118300 PMC11310283

[194] Veglia F Hashimoto A Dweep H Sanseviero E De Leo A Tcyganov E . Analysis of classical neutrophils and polymorphonuclear myeloid-derived suppressor cells in cancer patients and tumor-bearing mice. J Exp Med. (2021) 218:e20201803. doi: 10.1084/jem.20201803 33566112 PMC7879582

[195] Veglia F Tyurin VA Mohammadyani D Blasi M Duperret EK Donthireddy L . Lipid bodies containing oxidatively truncated lipids block antigen cross-presentation by dendritic cells in cancer. Nat Commun. (2017) 8:2122. doi: 10.1038/s41467-017-02186-9 29242535 PMC5730553

[196] Böttcher JP Bonavita E Chakravarty P Blees H Cabeza-Cabrerizo M Sammicheli S . Nk cells stimulate recruitment of cdc1 into the tumor microenvironment promoting cancer immune control. Cell. (2018) 172:1022–1037.e14. doi: 10.1016/j.cell.2018.01.004 29429633 PMC5847168

[197] Nieman KM Kenny HA Penicka CV Ladanyi A Buell-Gutbrod R Zillhardt MR . Adipocytes promote ovarian cancer metastasis and provide energy for rapid tumor growth. Nat Med. (2011) 17:1498–503. doi: 10.1038/nm.2492 22037646 PMC4157349

[198] Dirat B Bochet L Dabek M Daviaud D Dauvillier S Majed B . Cancer-associated adipocytes exhibit an activated phenotype and contribute to breast cancer invasion. Cancer Res. (2011) 71:2455–65. doi: 10.1158/0008-5472.Can-10-3323 21459803

[199] Balaban S Shearer RF Lee LS van Geldermalsen M Schreuder M Shtein HC . Adipocyte lipolysis links obesity to breast cancer growth: adipocyte-derived fatty acids drive breast cancer cell proliferation and migration. Cancer Metab. (2017) 5:1. doi: 10.1186/s40170-016-0163-7 28101337 PMC5237166

[200] Cai Z Liang Y Xing C Wang H Hu P Li J . Cancer‐associated adipocytes exhibit distinct phenotypes and facilitate tumor progression in pancreatic cancer. Oncol Rep. (2019) 42:2537–49. doi: 10.3892/or.2019.7365 31638193 PMC6826327

[201] Zoico E Darra E Rizzatti V Budui S Franceschetti G Mazzali G . Adipocytes Wnt5a mediated dedifferentiation: A possible target in pancreatic cancer microenvironment. Oncotarget. (2016) 7:20223–35. doi: 10.18632/oncotarget.7936 26958939 PMC4991449

[202] Gerst F Wagner R Oquendo MB Siegel-Axel D Fritsche A Heni M . What role do fat cells play in pancreatic tissue? Mol Metab. (2019) 25:1–10. doi: 10.1016/j.molmet.2019.05.001 31113756 PMC6600604

[203] Ziegler KM Considine RV True E Swartz-Basile DA Pitt HA Zyromski NJ . Adipocytes enhance murine pancreatic cancer growth via a hepatocyte growth factor (Hgf)-mediated mechanism. Int J Surg. (2016) 28:179–84. doi: 10.1016/j.ijsu.2016.03.002 26957017 PMC5433865

[204] Takehara M Sato Y Kimura T Noda K Miyamoto H Fujino Y . Cancer-associated adipocytes promote pancreatic cancer progression through Saa1 expression. Cancer Sci. (2020) 111:2883–94. doi: 10.1111/cas.14527 32535957 PMC7419047

[205] Winkler F Venkatesh HS Amit M Batchelor T Demir IE Deneen B . Cancer neuroscience: State of the field, emerging directions. Cell. (2023) 186:1689–707. doi: 10.1016/j.cell.2023.02.002 37059069 PMC10107403

[206] Zhang S Yuan L Lin P Yang G Zhou X Xu J . Cancer neuroscience: Signaling pathways and new therapeutic strategies for cancer. Signal Transduct Target Ther. (2026) 11:66. doi: 10.1038/s41392-025-02364-y 41724757 PMC12926231

[207] Stopczynski RE Normolle DP Hartman DJ Ying H DeBerry JJ Bielefeldt K . Neuroplastic changes occur early in the development of pancreatic ductal adenocarcinoma. Cancer Res. (2014) 74:1718–27. doi: 10.1158/0008-5472.Can-13-2050 24448244 PMC4036226

[208] Chen MM Gao Q Ning H Chen K Gao Y Yu M . Integrated single-cell and spatial transcriptomics uncover distinct cellular subtypes involved in neural invasion in pancreatic cancer. Cancer Cell. (2025) 43:1656–1676.e10. doi: 10.1016/j.ccell.2025.06.020 40680743

[209] Thiel V Renders S Panten J Dross N Bauer K Azorin D . Characterization of single neurons reprogrammed by pancreatic cancer. Nature. (2025) 640:1042–51. doi: 10.1038/s41586-025-08735-3 39961335 PMC12018453

[210] Xue M Zhu Y Jiang Y Han L Shi M Su R . Schwann cells regulate tumor cells and cancer-associated fibroblasts in the pancreatic ductal adenocarcinoma microenvironment. Nat Commun. (2023) 14:4600. doi: 10.1038/s41467-023-40314-w 37524695 PMC10390497

[211] Zhang B Guo X Huang L Zhang Y Li Z Su D . Tumour-associated macrophages and Schwann cells promote perineural invasion via paracrine loop in pancreatic ductal adenocarcinoma. Br J Cancer. (2024) 130:542–54. doi: 10.1038/s41416-023-02539-w 38135712 PMC10876976

[212] Wang L Liu Q Zhang Z Yang S Tang J Pan G . Prostaglandin E(2)-driven dedifferentiation of Schwann cells leads to perineural invasion in pancreatic ductal adenocarcinoma. Signal Transduct Target Ther. (2026) 11:122. doi: 10.1038/s41392-026-02648-x 41946682 PMC13056918

[213] Peng W Cao M Huang H Bai S Liu L Liang J . Cancer-associated fibroblast-derived sphingosine-1-phosphate activates a Mall-Sdc4 axis to facilitate perineural invasion in pancreatic cancer. Adv Sci (Weinh). (2026) 13:e75426. doi: 10.1002/advs.75426 42017444 PMC13335594

[214] Lakis V Chan NL Lyons RJ Blackburn N Nguyen TH Chang C . Spatial transcriptomics reveals novel mechanisms involved in perineural invasion in pancreatic ductal adenocarcinomas. Cancers. (2025) 17:852. doi: 10.3390/cancers17050852 40075699 PMC11899704

[215] Deborde S Gusain L Powers A Marcadis A Yu Y Chen CH . Reprogrammed Schwann cells organize into dynamic tracks that promote pancreatic cancer invasion. Cancer Discov. (2022) 12:2454–73. doi: 10.1158/2159-8290.Cd-21-1690 35881881 PMC9533012

[216] Zhang W-N He R Yang W-B Zhang Y Yuan Q-G Wang J . Autophagic Schwann cells promote perineural invasion mediated by the Ngf/Atg7 paracrine pathway in pancreatic cancer. J Exp Clin Cancer Res. (2022) 41:48. doi: 10.1186/s13046-021-02198-w 35109895 PMC8809009

[217] Arthur-Farraj P Latouche M Wilton DK Quintes S Chabrol E Banerjee A . C-Jun reprograms Schwann cells of injured nerves to generate a repair cell essential for regeneration. Neuron. (2012) 75:633–47. doi: 10.1016/j.neuron.2012.06.021 22920255 PMC3657176

[218] Gómez-Sánchez JA Carty L Iruarrizaga‐Lejarreta M Palomo-Irigoyen M Varela-Rey M Griffith M . Schwann cell autophagy, myelinophagy, initiates myelin clearance from injured nerves. J Cell Biol. (2015) 210:153–68. doi: 10.1083/jcb.201503019 PMC4494002 26150392

[219] Sah RP Sharma A Nagpal SJS Patlolla SH Sharma AK Kandlakunta H . Phases of metabolic and soft tissue changes in months preceding a diagnosis of pancreatic ductal adenocarcinoma. Gastroenterology. (2019) 156:1742–52. doi: 10.1053/j.gastro.2019.01.039 30677401 PMC6475474

[220] Naudin S Sampson JN Moore SC Albanes D Freedman ND Weinstein SJ . Lipidomics and pancreatic cancer risk in two prospective studies. Eur J Epidemiol. (2023) 38:783–93. doi: 10.1007/s10654-023-01014-3 37169992 PMC11152614

[221] Wang G Yao H Gong Y Lu Z Pang R Li Y . Metabolic detection and systems analyses of pancreatic ductal adenocarcinoma through machine learning, lipidomics, and multi-omics. Sci Adv. (2021) 7:eabh2724. doi: 10.1126/sciadv.abh2724 34936449 PMC8694594

[222] Wolrab D Jirásko R Cífková E Höring M Mei D Chocholousková M . Lipidomic profiling of human serum enables detection of pancreatic cancer. Nat Commun. (2022) 13:124. doi: 10.1038/s41467-021-27765-9 35013261 PMC8748654

[223] Peterka O Maccelli A Jirásko R Vaňková Z Idkowiak J Hrstka R . Hilic/Ms quantitation of low-abundant phospholipids and sphingolipids in human plasma and serum: Dysregulation in pancreatic cancer. Anal Chim Acta. (2023) 1288:342144. doi: 10.1016/j.aca.2023.342144 38220279

[224] Devasahayam Arokia Balaya R Sen P Grant CW Zenka RM Sappani M Lakshmanan J . An integrative multi-omics analysis reveals a multi-analyte signature of pancreatic ductal adenocarcinoma in serum. J Gastroenterol. (2024) 60:496–511. doi: 10.1007/s00535-024-02197-6 39666045 PMC13295322

[225] Peterka O Jirásko R Dolečková Z Dosoudilová M Bártl J Idkowiak J . Pilot study of screening method for pancreatic cancer using lipidomic profiling of plasma or serum. Commun Med. (2026) 6:127. doi: 10.1038/s43856-026-01445-5 41772143 PMC12954113

[226] Nakagawa N Hayashi M Kobayashi D Otsu T Sonohara F Kurimoto K . Exploration of lipid mediators for therapeutic monitoring of pancreatic cancer patients. Anticancer Res. (2025) 45:1343–53. doi: 10.21873/anticanres.17520 40155024

[227] Malhotra P Fyfe J Emmanouilidi A Casari I Mellett NA Huynh K . Oncogenic small extracellular vesicles enriched in sphingosine-1-phosphate play a crucial role in pancreatic cancer progression. Cell Signalling. (2025) 132:111775. doi: 10.1016/j.cellsig.2025.111775 40158707

[228] Amrutkar M Guttorm SJT Labori KJ Rootwelt H Elgstøen KBP Gladhaug IP . Reduced lipid metabolite abundance in human pancreatic cancer and matched serum samples following neoadjuvant Folfirinox treatment. Metabolomics. (2026) 22:18. doi: 10.1007/s11306-025-02388-z 41553593 PMC12816000

[229] Mayerle J Kalthoff H Reszka RC Kamlage B Peter E Schniewind B . Metabolic biomarker signature to differentiate pancreatic ductal adenocarcinoma from chronic pancreatitis. Gut. (2017) 67:128–37. doi: 10.1136/gutjnl-2016-312432 28108468 PMC5754849

[230] Mahajan UM Oehrle B Sirtl S Alnatsha A Goni E Regel I . Independent validation and assay standardization of improved metabolic biomarker signature to differentiate pancreatic ductal adenocarcinoma from chronic pancreatitis. Gastroenterology. (2022) 163:1407–22. doi: 10.1053/j.gastro.2022.07.047 35870514

[231] Mahajan UM Oehrle B Goni E Strobel O Kaiser JP Grützmann R . Validation of two plasma multimetabolite signatures for patients at risk of or with suspected pancreatic ductal adenocarcinoma (Metapac): A prospective, multicentre, investigator-masked, enrichment design, phase 4 diagnostic study. Lancet Gastroenterol Hepatol. (2025) 10:634–47. doi: 10.1016/s2468-1253(25)00056-1 40388948

[232] Siegel RL Kratzer TB Wagle NS Sung H Jemal A . Cancer statistics, 2026. Ca. (2026) 76:e70043. doi: 10.3322/caac.70043 41528114 PMC12798275

[233] Falchook GS Infante JR Arkenau H-T Patel MR Dean E Borazanci EH . First-in-human study of the safety, pharmacokinetics, and pharmacodynamics of first-in-class fatty acid synthase inhibitor Tvb-2640 alone and with a taxane in advanced tumors. EClinicalMedicine. (2021) 34:100797. doi: 10.1016/j.eclinm.2021.100797 33870151 PMC8040281

[234] Fako V Wu X Pflug BR Liu J-Y Zhang J-T . Repositioning proton pump inhibitors as anticancer drugs by targeting the thioesterase domain of human fatty acid synthase. J Med Chem. (2014) 58:778–84. doi: 10.1021/jm501543u 25513712 PMC4306520

[235] ClinicalTrials.gov . High dose omeprazole in patients with pancreatic cancer. ClinicalTrialsgov. (2026).

[236] Sokołowska E Presler M Goyke E Milczarek R Świerczyṅski J Śledziński T . Orlistat reduces proliferation and enhances apoptosis in human pancreatic cancer cells (Panc-1). Anticancer Res. (2017) 37:6321–7. doi: 10.21873/anticanres.12083 29061815

[237] Wang X Tian W . Green tea epigallocatechin gallate: A natural inhibitor of fatty-acid synthase. Biochem Biophys Res Commun. (2001) 288:1200–6. doi: 10.1006/bbrc.2001.5923 11700039

[238] ClinicalTrials.gov . Gemcitabine hydrochloride, paclitaxel albumin-stabilized nanoparticle formulation, metformin hydrochloride, and a standardized dietary supplement in treating patients with pancreatic cancer that cannot be removed by surgery. ClinicalTrialsgov. (2026).

[239] Liu Q Ge W Wang T Lan J Martínez-Jarquín S Wolfrum C . High-throughput single-cell mass spectrometry reveals abnormal lipid metabolism in pancreatic ductal adenocarcinoma. Angew Chem Int Ed Engl. (2021) 60:24534–42. doi: 10.1002/anie.202107223 34505339 PMC8597026

[240] Rebelo A Kleeff J Sunami Y . Cholesterol metabolism in pancreatic cancer. Cancers. (2023) 15:5177. doi: 10.3390/cancers15215177 37958351 PMC10650553

[241] Hong JY Nam EM Lee J Park J-O Lee S-C Song SY . Randomized double-blinded, placebo-controlled phase Ii trial of simvastatin and gemcitabine in advanced pancreatic cancer patients. Cancer Chemotherapy Pharmacol. (2013) 73:125–30. doi: 10.1007/s00280-013-2328-1 24162380

[242] Tamburrino D Crippa S Partelli S Archibugi L Arcidiacono PG Falconi M . Statin use improves survival in patients with pancreatic ductal adenocarcinoma: A meta-analysis. Digestive liver Dis Off J Ital Soc Gastroenterol Ital Assoc For Study Liver. (2020) 52:392–9. doi: 10.1016/j.dld.2020.01.008 32113888

[243] Karbowska E Świeczkowski D Gąsecka A Pruc M Safiejko K Ladny JR . Statins and the risk of pancreatic cancer: A systematic review and meta-analysis of 2,797,186 patients. Cardiol J. (2022) 31:243–50. doi: 10.5603/CJ.a2022.0014 PMC11076033 35373328

[244] Budillon A Leone A Passaro E Silvestro L Foschini F Iannelli F . Randomized phase 2 study of valproic acid combined with simvastatin and gemcitabine/nab-paclitaxel-based regimens in untreated metastatic pancreatic adenocarcinoma patients: The Vespa trial study protocol. BMC Cancer. (2024) 24:1167. doi: 10.1186/s12885-024-12936-w 39300376 PMC11414294

[245] ClinicalTrials.gov . Cholesterol disruption in combination with the standard of care in patients with advanced pancreatic adenocarcinoma. ClinicalTrialsgov. (2026).

[246] Ortiz-Placín C Castillejo-Rufo A Estarás M González A . Membrane lipid derivatives: Roles of arachidonic acid and its metabolites in pancreatic physiology and pathophysiology. Molecules. (2023) 28:4316. doi: 10.3390/molecules28114316 PMC10254454 37298790

[247] Dragovich T Burris HA Loehrer PJ Von Hoff DD Chow S Stratton SP . Gemcitabine plus celecoxib in patients with advanced or metastatic pancreatic adenocarcinoma: Results of a phase Ii trial. Am J Clin Oncol. (2008) 31:157–62. doi: 10.1097/coc.0b013e31815878c9 18391600

[248] Kirane A Toombs JE Ostapoff KT Carbon JG Zaknoen SL Braunfeld J . Apricoxib, a novel inhibitor of Cox-2, markedly improves standard therapy response in molecularly defined models of pancreatic cancer. Clin Cancer Res Off J Am Assoc For Cancer Res. (2012) 18:5031–42. doi: 10.1158/1078-0432.ccr-12-0453 22829202 PMC3777527

[249] Apricot-P (Apricoxib in Combination Oncology Treatment - Pancreas): Phase 2 study of the efficacy and safety of Apricoxib in combination with gemcitabine and erlotinib in the treatment of patients with advanced pancreatic cancer [Internet (2008). Available online at: https://clinicaltrials.gov/study/NCT00709826 (Accessed October 19, 2026).

[250] Hong DS Parikh AR Shapiro GI Varga A Naing A Meric-Bernstam F . First-in-human phase I study of immunomodulatory E7046, an antagonist of Pge2-receptor E-type 4 (Ep4), in patients with advanced cancers. J Immunother Cancer. (2020) 8:e000222. doi: 10.1136/jitc-2019-000222 32554609 PMC7304851

[251] He J Lin X Meng F Zhao Y Wang W Zhang Y . A novel small molecular prostaglandin receptor Ep4 antagonist, L001, suppresses pancreatic cancer metastasis. Molecules. (2022) 27:1209. doi: 10.3390/molecules27041209 35208999 PMC8879074

[252] Hu W Liu S Zhu K Li W Yang Z-J Yang Q . The Alox5 inhibitor zileuton regulates tumor-associated macrophage M2 polarization by Jak/Stat and inhibits pancreatic cancer invasion and metastasis. Int Immunopharmacol. (2023) 121:110505. doi: 10.1016/j.intimp.2023.110505 37348233

[253] Ferry DR Deakin M Baddeley J Daryanani S Bramhall S Anderson D . A phase II study of the 5-lipoxygenase inhibitor, CV6504, in advanced pancreatic cancer: Correlation of clinical data with pharmacokinetic and pharmacodynamic endpoints. Ann Oncol Off J Eur Soc For Med Oncol. (2000) 11:1165–70. doi: 10.1023/a:1008303715515 11061613

[254] Shinoda S Nakamura N Inoko K Sato-Dahlman M Carmella SG Hecht SS . Inhibition of fatty acid binding protein suppresses pancreatic cancer progression and metastasis. Sci Rep. (2025) 15:26084. doi: 10.1038/s41598-025-11271-9 40681747 PMC12274511

[255] Wang K Zhang Z Wang M Cao X Qi J Wang D . Role of GRP78 inhibiting artesunate-induced ferroptosis in KRAS mutant pancreatic cancer cells. Drug Des Dev Ther. (2019) 13:2135–44. doi: 10.2147/dddt.s199459 31456633 PMC6620771

[256] Li C Zhang Y Liu J Kang R Klionsky DJ Tang D . Mitochondrial DNA stress triggers autophagy-dependent ferroptotic death. Autophagy. (2021) 17:948–60. doi: 10.1080/15548627.2020.1739447 32186434 PMC8078708

[257] Karasic TB O'Hara MH Loaiza-Bonilla A Reiss KA Teitelbaum U Borazanci EH . Effect of gemcitabine and nab-paclitaxel with or without hydroxychloroquine on patients with advanced pancreatic cancer: A phase 2 randomized clinical trial. JAMA Oncol. (2019) 5:993–8. doi: 10.1001/jamaoncol.2019.0684 31120501 PMC6547080

[258] Zeh HJ Bahary N Boone BA Singhi AD Miller-Ocuin JL Normolle DP . A randomized phase II preoperative study of autophagy inhibition with high-dose hydroxychloroquine and gemcitabine/nab-paclitaxel in pancreatic cancer patients. Clin Cancer Res Off J Am Assoc For Cancer Res. (2020) 26:3126–34. doi: 10.1158/1078-0432.ccr-19-4042 32156749 PMC8086597

[259] Cheng C Hu J Mannan R He T Bhattacharyya R Magnuson B . Targeting PIKfyve-driven lipid metabolism in pancreatic cancer. Nature. (2025) 642:776–84. doi: 10.1038/s41586-025-08917-z 40269157 PMC12176661

[260] Cao R Feng Z Mo J Wu J Li J Li W . Pharmacological inhibition of SREBP1 suppresses pancreatic cancer growth via inducing GPX4-mediated ferroptosis. Cell Signalling. (2024) 124:111381. doi: 10.1016/j.cellsig.2024.111381 39243918

[261] Mouhid L Gómez de Cedrón M García-Carrascosa E Reglero G Fornari T Ramírez de Molina A . Yarrow supercritical extract exerts antitumoral properties by targeting lipid metabolism in pancreatic cancer. PloS One. (2019) 14:e0214294. doi: 10.1371/journal.pone.0214294 30913248 PMC6435158

[262] Karaboga H Huang W Srivastava SK Widmann S Addanki S Gamage K . Screening of focused compound library targeting liver X receptors in pancreatic cancer identified ligands with inverse agonist and degrader activity. ACS Chem Biol. (2020) 15:2916–28. doi: 10.1021/acschembio.0c00546 33074669

[263] Gao Y Jia Z Kong X Li Q Chang DZ Wei D . Combining betulinic acid and mithramycin A effectively suppresses pancreatic cancer by inhibiting proliferation, invasion and angiogenesis. Cancer Res. (2011) 71:5182–93. doi: 10.1158/0008-5472.can-10-2016 21673052 PMC3245664

[264] A phase 1, first-in-human study of escalating doses of oral TVB-2640 in patients with solid tumors (2014). Available online at: https://clinicaltrials.gov/study/NCT02223247 (Accessed October 19, 2026).

[265] Wei R Cortez Penso NE Hackman RM Wang Y Mackenzie GG . Epigallocatechin-3-gallate (EGCG) suppresses pancreatic cancer cell growth, invasion, and migration partly through the inhibition of Akt pathway and epithelial–mesenchymal transition: Enhanced efficacy when combined with gemcitabine. Nutrients. (2019) 11:1856. doi: 10.3390/nu11081856 31405071 PMC6722696

[266] Hoy AJ Nagarajan SR Butler LM . Tumour fatty acid metabolism in the context of therapy resistance and obesity. Nat Rev Cancer. (2021) 21:753–66. doi: 10.1038/s41568-021-00388-4 34417571

[267] Randomized double-blinded, placebo-controlled phase II trial of simvastatin and gemcitabine in advanced pancreatic cancer patients (2009). Available online at: https://clinicaltrials.gov/study/NCT00944463 (Accessed October 19, 2026). 10.1007/s00280-013-2328-1 24162380

[268] Karavias D Thomas P Koh A Irving GRB Navarro AP Cameron IC . Statin therapy does not influence the outcome of patients undergoing surgery for pancreatic cancer. Anz J Surg. (2020) 90:1671–6. doi: 10.1111/ans.15600 31845479

[269] A phase II trial of gemcitabine and celecoxib as first-line treatment for patients with advanced metastatic pancreatic cancer (2003). Available online at: https://clinicaltrials.gov/study/NCT00068432 (Accessed October 19, 2026).

[270] Kuda O Pietka TA Demianova Z Kudova E Cvacka J Kopecky J . Sulfo-N-succinimidyl oleate (SSO) inhibits fatty acid uptake and signaling for intracellular calcium via binding CD36 lysine 164: SSO also inhibits oxidized low density lipoprotein uptake by macrophages. J Biol Chem. (2013) 288:15547–55. doi: 10.1074/jbc.M113.473298 23603908 PMC3668716

[271] Divakaruni AS Hsieh WY Minarrieta L Duong TN Kim KKO Desousa BR . Etomoxir inhibits macrophage polarization by disrupting CoA homeostasis. Cell Metab. (2018) 28:490–503.e7. doi: 10.1016/j.cmet.2018.06.001 30043752 PMC6125190

[272] Randomized phase II trial of pre-operative gemcitabine and nab paclitacel with or with out hydroxychloroquine (2013). Available online at: https://clinicaltrials.gov/study/NCT01978184 (Accessed October 19, 2026).

[273] A phase II multicenter trial of ESK981 in patients with select solid tumors (2023). Available online at: https://clinicaltrials.gov/study/NCT05988918 (Accessed October 19, 2026).

[274] Yu S Wang L Che D Zhang M Li M Naito M . Targeting CRABP-II overcomes pancreatic cancer drug resistance by reversing lipid raft cholesterol accumulation and Akt survival signaling. J Exp Clin Cancer Res CR. (2022) 41:88. doi: 10.21203/rs.3.rs-929878/v1 35260193 PMC8903155

[275] Cao Y Wang X Liu Y Liu P Qin J Zhu Y . BHLHE40 inhibits ferroptosis in pancreatic cancer cells via upregulating SREBF1. Adv Sci. (2024) 11:e2306298. doi: 10.1002/advs.202306298 38064101 PMC10870036

[276] Markosyan N Kim I Arora C Quinones-Ware L Joshi N Cheng N . Pivotal roles for cancer cell–intrinsic mPGES-1 and autocrine EP4 signaling in suppressing antitumor immunity. JCI Insight. (2024) 9:e178644. doi: 10.1172/jci.insight.178644 39298269 PMC11601572

